# Assembling contigs in draft genomes using reversals and block-interchanges

**DOI:** 10.1186/1471-2105-14-S5-S9

**Published:** 2013-04-10

**Authors:** Chi-Long Li, Kun-Tze Chen, Chin Lung Lu

**Affiliations:** 1 Department of Computer Science, National Tsing Hua University, Hsinchu 30013, Taiwan

## Abstract

The techniques of next generation sequencing allow an increasing number of draft genomes to be produced rapidly in a decreasing cost. However, these draft genomes usually are just partially sequenced as collections of unassembled contigs, which cannot be used directly by currently existing algorithms for studying their genome rearrangements and phylogeny reconstruction. In this work, we study the one-sided block (or contig) ordering problem with weighted reversal and block-interchange distance. Given a partially assembled genome *π *and a completely assembled genome *σ*, the problem is to find an optimal ordering to assemble (i.e., order and orient) the contigs of *π *such that the rearrangement distance measured by reversals and block-interchanges (also called generalized transpositions) with the weight ratio 1:2 between the assembled contigs of *π *and *σ *is minimized. In addition to genome rearrangements and phylogeny reconstruction, the one-sided block ordering problem particularly has a useful application in genome resequencing, because its algorithms can be used to assemble the contigs of a draft genome *π *based on a reference genome *σ*. By using permutation groups, we design an efficient algorithm to solve this one-sided block ordering problem in $O\left(\delta n\right)$ time, where *n *is the number of genes or markers and *δ *is the number of used reversals and block-interchanges. We also show that the assembly of the partially assembled genome can be done in $O\left(n\right)$ time and its weighted rearrangement distance from the completely assembled genome can be calculated in advance in $O\left(n\right)$ time. Finally, we have implemented our algorithm into a program and used some simulated datasets to compare its accuracy performance to a currently existing similar tool, called SIS that was implemented by a heuristic algorithm that considers only reversals, on assembling the contigs in draft genomes based on their reference genomes. Our experimental results have shown that the accuracy performance of our program is better than that of SIS, when the number of reversals and transpositions involved in the rearrangement events between the complete genomes of *π *and *σ *is increased. In particular, if there are more transpositions involved in the rearrangement events, then the gap of accuracy performance between our program and SIS is increasing.

## Background

The techniques of next generation sequencing have greatly advanced in the past decade [1-3], which allows an increasing number of draft genomes to be produced rapidly in a decreasing cost. Usually, these draft genomes are partially sequenced, leading to their published genomes as collections of unassembled contigs (short for contiguous fragments). These draft genomes in contig form, however, can not be used immediately in some bioinformatics applications, such as the study of genome rearrangements, which requires the completely assembled genomes to calculate their rearrangement distances [4]. To adequately address this issue, Gaul and Blanchette [5] introduced and studied the so-called block ordering problem defined as follows. Given two partially assembled genomes, with each representing as an unordered set of blocks, the *block ordering problem *is to assemble (i.e., order and orient) the blocks of the two genomes such that the distance of genome rearrangements between the two assembled genomes is minimized. The blocks mentioned above are the contigs, each of which can be represented by an ordered list of genes or markers. In their work [5], Gaul and Blanchette proposed a linear-time algorithm to solve the block ordering problem if the problem is further simplified to maximize the number of cycles in the breakpoint graph corresponding to the assembled genomes. The rationale behind this modification is totally based on a result obtained by Bourque and Pevzner [6], showing that the reversal distance between two assembled genomes can be approximated well by maximizing the number of cycles in their corresponding breakpoint graph. Actually, in addition to the number of cycles, the number of hurdles, as well as the presence of a fortress or not, is also important and needed for determining the actual reversal distance [7]. Therefore, it is still a challenge to efficiently solve the block ordering problem by optimizing the true rearrangement distance.

In the literature, many different kinds of genome rearrangements have been extensively studied [4], such as reversal (also called inversion), transposition and block-interchange (also called generalized transposition), translocation, fusion and fission. Reversal affects a segment on a chromosome by reversing this segment as well as exchanging its strands. Transposition rearranges a chromosome by interchanging its two adjacent and nonoverlapping segments. Block-interchange is a generalized transposition that exchanges two nonoverlapping but not necessarily adjacent segments on a chromosome. Translocation acts on two chromosomes by exchanging their the end fragments. Fusion is a special translocation that joins two chromosomes into one and fission is also a special translocation that splits a chromosome into two. In this study, we consider a variant of the block ordering problem, in which one of the two input genomes is still partially assembled but the other is completely assembled, with optimizing the genome rearrangement distance measured by weighted reversals and block-interchanges, whose weights are 1 and 2, respectively. For distinguishing this special block ordering problem from the original one, we call it as *one-sided block *(*or contig*) *ordering problem*. In fact, an efficient algorithm to solve the one-sided block ordering problem has a useful application in genome resequencing [8,9], because the reference genome for resequencing organisms can serve as the completely assembled genome in the one-sided block ordering problem and the contigs of partially assembled resequencing genome can then be assembled together into one or several scaffolds based on the reference genome. From this respect, the one-sided block ordering problem can be considered as a kind of *contig scaffolding *(*or assembly*) *problem *that aims to use genome rearrangements to create contig scaffolds for a draft genome based on a reference genome.

Currently, several contig scaffolding tools based on the reference genomes have been developed, such as Projector 2 [10], OSLay [11], ABACAS [12], Mauve Aligner [13], fillScaffolds [14], r2cat [15] and SIS [16]. Among these contig scaffolding tools, both SIS and fillScaffolds use the concept of genome rearrangements to generate contig scaffolds for a draft genome. SIS deals with only reversals, while in addition to reversals, fillScaffolds considers other rearrangements, such as transpositions and translocations (including fissions and fusions). Basically, SIS was dedicated to creating the contig scaffolds for prokaryotic draft genomes by heuristically searching for their inversion signatures, where an *inversion signature *is a pair of adjacent genes or markers appearing along a contig such that they form a breakpoint and are also located in different transcriptional strands. As for fillScaffolds, it used the traditional technique of breakpoint graphs to assemble the contigs of draft genomes. In the study by Dias and colleagues [16], they have used real prokaryotic draft genomes to demonstrate that SIS had the best overall accuracy performance when compared to the other tools we mentioned above.

In this study, we utilize permutation groups in algebra, instead of the breakpoint graphs used by Gaul and Blanchette [5], to design an efficient algorithm, whose time complexity is $O\left(\delta n\right)$, for solving the one-sided block ordering problem with weighted reversal and block-interchange distance, where *n *is the number of genes or markers and *δ *is the number of reversals and block-interchanges used to transform the assembly of the partially assembled genome (i.e., draft genome) into the completely assembled genome (i.e., reference genome). In particular, we also show that the assembly of the partially assembled genome can be done in $O\left(n\right)$ time and its weighted reversal and block-interchange distance from the completely assembled genome can be calculated in advance in $O\left(n\right)$ time. In addition, we have implemented our algorithm into a program and used some simulated datasets to compare its accuracy performance to SIS on assembling the contigs in the draft genomes based on their reference genomes. Our experimental results have shown that the averaged normalized contig mis-join errors of our program are lower than those of SIS, when the number of reversals and transpositions involved in the rearrangement events between the complete genomes of the partially and completely assembled organisms is increased. In particular, if there are more transpositions involved in the rearrangement events, then the gap of accuracy performance between our program and SIS is increasing.

## Preliminaries

### One-sided block ordering problem

In the following, we dedicate ourselves to linear, uni-chromosomal genomes. With a slight modification, however, our algorithmic result can still apply to circular, uni-chromosomal genomes, or to multi-chromosomal genomes with linear or circular chromosomes in a chromosome-by-chromosome manner. Once completely assembled, a uni-chromosomal genome can be represented by a signed permutation of *n *integers between 1 and *n*, with each integer representing a gene or marker and its associated sign indicating the strandedness of the corresponding gene or marker. If the genome is partially assembled, then it will be represented by an unordered set of blocks, where a block *B *of size *k*, denoted by *B *= [*b*_1_, *b*_2_, ..., *b_k_*], is an ordered list of *k *signed integers. Let $\bar{B}=\left[-b_{k},\;-b_{k-1},\;\ldots ,\;-b_{1}\right]$ denote the *reverse *of *B*. Given an unordered set of *m *blocks, say $B=\left\{B_{1},\;B_{2},\;\ldots ,\;B_{m}\right\}$, corresponding to a partially assembled genome, an *ordering *(or *assembly*) of  B is an ordered list of *m *blocks in which each block *B_i _*or its reverse $\bar{B_{i}}$ appears exactly once, where 1 ≤ *i *≤ *m*. For instance, suppose that $B=\left\{B_{1},B_{2},B_{3}\right\}=\left\{\left[1,4\right],\left[3,2\right],\left[-5,6\right]\right\}$. Then (*B*_1_, *B*_3_, *B*_2_) = ([1, 4], [-5, 6], [3, 2]) and (*B*_1_, -*B*_3_, *B*_2_) = ([1, 4], [-6, 5], [3, 2]) are two orderings of  B. Basically, each ordering of  B*induces *(or *defines*) a signed permutation of size *n*, which is obtained by concatenating the blocks in this ordered list. For instance, the ordering (*B*_1_, *B*_3_, *B*_2_) in the above exemplified  B induces the signed permutation (1, 4, -5, 6, 3, 2), which simply is denoted by *B*_1 _⊙ *B*_3 _⊙ *B*_2_. Clearly, the permutation induced by an ordering of  B corresponds to an assembly of the blocks in  B. Now, the one-sided block ordering problem we study in this paper is formally defined as follows:

### One-sided block ordering problem with reversal and block-interchange distance

**Input: **A partially assembled genome *π *and a completely assembled genome *σ*.

**Output: **Find an ordering of *π *such that the rearrangement distance measured by reversals and block-interchanges with the weight ratio 1:2 between the permutation induced by the ordering of *π *and *σ *is minimized.

As discussed in our previous study [17], it is biologically meaningful to assign twice the weight to block-interchanges than to reversals, due to the observation from the biological data that transpositions occur with about half the frequency of reversals [18].

### Permutation groups

Permutation groups have been proven to be a very useful tool in the studies of genome rearrangements [17]. Below, we recall some useful definitions, notations and properties borrowed form our previous work [17]. Basically, given a set *E *= {1, 2, ..., *n*}, a *permutation *is defined to be a one-to-one function from *E *into itself and usually expressed as a product of cycles in the study of genome rearrangements. For instance, *π *= (1)(3, 2) is a product of two cycles to represent a permutation of *E *= {1, 2, 3} and means that *π*(1) = 1*, π*(2) = 3 and *π*(3) = 2. The elements in a cycle can be arranged in any cyclic order and hence the cycle (3, 2) in the permutation *π *exemplified above can be rewritten as (2, 3). Moreover, if the cycles in a permutation are all disjoint (i.e., no common element in any two cycles), then the product of these cycles is called the *cycle decomposition *of the permutation. In fact, a permutation in the cycle decomposition can be used to model a genome containing several circular chromosomes, with each disjoint cycle representing a circular chromosome. Notice that in the rest of this article, we say "cycle in a permutation" to mean "cycle in the cycle decomposition of this permutation" for simplicity, unless otherwise specified. A cycle with *k *elements is further called a *k*-*cycle*. In convention, the 1-cycles in a permutation are not written explicitly since their elements are *fixed *in the permutation. For instance, the above exemplified permutation *π *can be written as *π *= (2, 3). If the cycles in a permutation are all 1-cycles, then this permutation is called an *identify permutation *and denoted by **1**. Suppose that *α *and *β *are two permutations of *E*. Then their product *αβ*, also called their *composition*, defines a permutation of *E *satisfying *αβ*(*x*) = *α*(*β*(*x*)) for all x∈E. If both α and *β *are disjoint, then *αβ *= *βα*. If *αβ *= **1**, then *α *is called the *inverse *of *β*, denoted by *β*^-1^, and vice versa. Moreover, the *conjugation *of *β *by *α*, denoted by *α *· *β*, is defined to be the permutation $\alpha \beta {\alpha^{-}}^{\text{1}}$. It can be verified that if *y *= *β*(*x*), then *α*(*y*) = *αβ*(*x*) = *αβα*^-1^*α*(*x*) = *α *· *β*(*α*(*x*)). Hence, *α *· *β *can be obtained from *β *by just changing its element *x *with *α*(*x*). In other words, if *β *= (*b*_1_, *b*_2_, ..., *b_k_*), then *α *· *β *= (*α*(*b*_1_), *α*(*b*_2_), ..., *α*(*b_k_*)).

It is a fact that every permutation can be expressed into a product of 2-cycles, in which 1-cycles are still written implicitly. Given a permutation *α *of *E*, its *norm*, denoted by ||*α*||, is defined to be the minimum number, say *k*, such that *α *can be expressed as a product of *k *2-cycles. In the cycle decomposition of *α*, let *n_c_*(*α*) denote the number of its disjoint cycles, notably including the 1-cycles not written explicitly. Given two permutations *α *and *β *of *E*, *α *is said to *divide β*, denoted by *α|β*, if and only if ||*βα*^-1^|| = ||*β*|| *- *||*α*||. In our previous work [17], it has been shown that ||*α*|| = *|E| - n_c_*(*α*) and for any *k *elements in *E*, say *a*_1_, *a*_2_, ..., *a_k_*, they all appear in a cycle of *α *in the ordering of *a*_1_, *a*_2_, ..., *a_k _*if and only if (*a*_1_, *a*_2_, ..., *a_k_*) *| α*.

Let *α *= (*a*_1_*, a*_2_) be a 2-cycle and *β *be an arbitrary permutation of *E*. If *α|β*, that is, both *a*_1 _and *a*_2 _appear in the same cycle of *β*, then the composition *αβ*, as well as *βα*, has the effect of fission by breaking this cycle into two smaller cycles. For instance, let *α *= (1, 3) and *β *= (1, 2, 3, 4). Then *α|β*, since both 1 and 3 are in the cycle (1, 2, 3, 4), and *αβ *= (1, 2)(3, 4) and *βα *= (4, 1)(2, 3). On the other hand, if α∤β, that is, *a*_1 _and *a*_2 _appear in different cycles of *β*, then *αβ*, as well as *βα*, has the effect of fusion by joining the two cycles into a bigger cycle. For example, if *α *= (1, 3) and *β *= (1, 2)(3, 4), then α∤β and, as a result, *αβ *= (1, 2, 3, 4) and *βα *= (2, 1, 4, 3).

### A model for representing DNA molecules

As mentioned before, a permutation in the form of the cycle decomposition can be used to model a genome containing multiple chromosomes (or a chromosome with multiple contigs), with each cycle representing a chromosome (or contig). To facilitate modelling the rearrangement of reversals using the permutation groups, however, we need to use two cycles to represent a chromosome, with one cycle representing a strand of the chromosome and the other representing the complementary strand. For this purpose, we first let *E *= {-1, 1, -2, 2, ..., *-n, -n*} and Γ = (1, -1)(2, -2) ... (*n, -n*). We then use an *admissible *cycle, which is a cycle containing no *i *and its opposite *-i *simultaneously for some i∈E, to represent a chromosomal strand, say *π*^+^, and use *π*^- ^= Γ · (*π*^+^)^-1^, which is the *reverse complement *of *π*^+^, to represent the opposite strand of *π*^+^. As demonstrated in our previous work [17], it is useful to represent a double stranded chromosome *π *by the product of its two strands *π*^+ ^and *π*^-^, that is, $\pi =\pi^{+}\pi^{-}=\pi^{-}\pi^{+}$, because a reversal (respectively, block-interchange) acting on this DNA molecule can be mimicked by multiplying two (respectively, four) 2-cycles with *π*, as described in the following lemmas.

**Lemma 1 (**[17]**) ***Let π *= *π*^+^*π*^- ^*denote a double stranded DNA and let x and y be two elements in E. If *(x,y)∤π, *that is, x and y are in the different strands of π, then the effect of *(*π*Γ(*y*), *π*Γ(*x*))(*x*, *y*)*π is a reversal acting on π*.

**Lemma 2 (**[17]**) ***Let π *= *π*^+^*π*^- ^*denote a double stranded DNA and let u, v, x and y be four elements in E. If *(*x, u, y, v*)*|π, that is, x, u, y and v appear in the same strand of π in this order, then the effect of *(*π*Γ(*v*)*, π*Γ(*u*)) (*π*Γ(*y*)*, π*Γ(*x*)) (*u, v*)(*x, y*)*π is a block-interchange acting on π*.

Moreover, as described in the following lemma, we have shown in [17] that given two different DNA molecules *π *and *σ*, every cycle *α *in (the cycle decomposition of) *σπ*^-1 ^always has a *mate *cycle (*π*Γ) · *α*^-1 ^that also appears in *σπ*^-1^. In fact, *α *and (*π*Γ) · *α*^-1 ^in *σπ*^-1 ^are each other's mate cycle.

**Lemma 3 (**[17]**) ***Let π and σ be two different double-stranded DNA molecules. If α is a cycle in σπ*^-1^, *then *(*π*Γ) · *α*^-1 ^*is also a cycle in σπ*^-1^.

### An efficient algorithm for the one-sided block ordering problem

To clarify our algorithm, we start with defining some notations. Let *α *denote an arbitrary linear DNA molecule (or contig). As mentioned previously, it is represented by the product of its two strands *α*^+ ^and *α^-^*, that is, *α *= *α*^+^*α^-^*. If *α *contains *k *genes (or markers), we also denote its *α*^+ ^by (*α*^+^[1]*, α*^+^[2], ..., *α*^+^[*k*]), where *α*^+^[*i*] is the *i*-th gene in *α*, and its *α*^- ^by (*α^- ^*[1]*, α^-^*[2], ..., *α^-^*[*k*]). By convention, *α*^+^[1] and *α^-^*[1] are called as *tails *of *α*. Let *π *= *π*_1_*π*_2 _... *π_m _*be a linear, uni-chromosomal genome that is partially assembled into *m *contigs *π*_1_, *π*_2_, ..., *π_m_*, each with *n_i _*genes, and σ = (1, 2*, ..., n*) be a linear, uni-chromosomal genome that is assembled completely. Let *C *= {*c_k _*= *n *+ *k *+ 1: 0 ≤ *k *≤ 2*m - *1} ∪ {*-c_k _*= *-n - k - *1: 0 ≤ *k *≤ 2*m - *1} be a set of 4*m *distinct integers, called *caps*, which are different from those genes in *E*. Let $\hat{E}=E\;\cup \;C$ and $\hat{\text{Γ}}=\left(1,\;-1\right)\left(2,\;-2\right)\;\ldots \;\left(n+2m,\;-n-2m\right)$. For the purpose of designing our algorithm later, we add four caps $c_{2\left(i-1\right)},c_{2\left(i-1\right)+1},-c_{2\left(i-1\right)}$ and *-c*_2(*i-*1)+1 _to the ends of each contig *π_i_*, where 1 ≤ *i *≤ *m*, leading to a capping contig ${\hat{\pi }}_{i}$ with ${\hat{\pi }}_{i}^{+}\left[1\right]=c_{2\left(i-1\right)},\;{\hat{\pi }}_{i}^{+}\left[j\right]=\pi_{i}^{+}\left[j-1\right]$, for $2\leq j\leq n_{i}+1,\;{\hat{\pi }}_{i}^{+}\left[n_{i}+2\right]=c_{2\left(i-1\right)+1},\;{\hat{\pi }}_{i}^{-}\left[1\right]=\hat{\text{Γ}}\left(c_{2\left(i-1\right)+1}\right),\;{\hat{\pi }}_{i}^{-}\left[j\right]=\pi_{i}^{-}\left[j-1\right]$ for 2 ≤ *j *≤ *n_i _*+ 1, and ${\hat{\pi }}_{i}^{-}\left[n_{i}+2\right]=\hat{\text{Γ}}\left(c_{2\left(i-1\right)}\right)$. Moreover, we insert *m*-1 dummy contigs without any genes (i.e., null contigs) *σ*_2_, *σ*_3_, ..., *σ_m _*into *σ*, where the original contig in *σ *becomes *σ*_1 _now, and add four caps *c*_2(*i-*1)_*, c*_2(*i-*1)+1_*, -c*_2(*i-*1) _and *-c*_2(*i-*1)+1 _to the ends of each contig *σ_i _*to obtain a capping contig ${\hat{\sigma }}_{i}$, where ${\hat{\sigma }}_{i}^{+}\left[1\right]=c_{2\left(i-1\right)},{\hat{\sigma }}_{i}^{+}\left[j\right]=\sigma_{i}^{+}\left[j-1\right]$ for $2\leq j\leq n_{i}+1,{\hat{\sigma }}_{i}^{+}\left[n_{i}+2\right]=c_{2\left(i-1\right)+1},\;{\hat{\sigma }}_{i}^{-}\left[1\right]=\hat{\text{Γ}}\left(c_{2\left(i-1\right)+1}\right),{\hat{\sigma }}_{i}^{-}\left[j\right]=\sigma_{i}^{-}\left[j-1\right]$ for 2 ≤ *j *≤ *n_i _*+ 1, and ${\hat{\sigma }}_{i}^{-}\left[n_{i}+2\right]=\hat{\text{Γ}}\left(c_{2\left(i-1\right)}\right)$. Notice that the purpose of adding caps to the ends of the contigs is to serve as delimiters when we use permutation groups to model translocations of multiple contigs later. We denote the capping *π *and *σ *by $\hat{\pi }$ and $\hat{\sigma }$, respectively. To distinguish the four caps in a capping contig, say ${\hat{\pi }}_{i}$, we call the left caps ${\hat{\pi }}_{i}^{+}\left[1\right]$ and ${\hat{\pi }}_{i}^{-}\left[1\right]$ as 5' caps and the right caps ${\hat{\pi }}_{i}^{+}\left[n_{i}+2\right]$ and ${\hat{\pi }}_{i}^{-}\left[n_{i}+2\right]$ as 3' caps.

Given an integer *x *in $\hat{E}$ that is contained in a contig *α *= *α*^+^*α^- ^*with *k *genes (or markers), we define a function **char**(*x*, *$\hat{\alpha }$*) below to represent the character of *x *in the capping contig $\hat{\alpha }={\hat{\alpha }}^{+}{\hat{\alpha }}^{-}$ that is obtained by adding four caps from *C *to the ends of *α*.

$$\text{char}\left(x,\;\hat{\alpha }\right)=\left\{\begin{array}{cc} \text{C}5, & \text{if}\;x={\hat{\alpha }}^{+}\left[1\right]\;\text{or}\;x={\hat{\alpha }}^{-}\left[1\right]\\ & \left(\text{that}\;\text{is},\;x\;\text{is}\;\text{a}\;5^{\prime }\;\text{cap}\;\text{in}\;\hat{\alpha }\right).\\ \text{C}3, & \text{if}\;k\neq 0\;\text{and}\;\left(x={\hat{\alpha }}^{+}\left[k+2\right]\;\text{or}\;x={\hat{\alpha }}^{-}\left[k+2\right]\right)\\ & \left(\text{that}\;\text{is},\;\alpha \;\text{is}\;\text{not}\;\text{null}\;\text{and}\;x\;\text{is}\;\text{a}\;3^{\prime }\;\text{cap}\;\text{in}\;\hat{\alpha }\right).\\ \text{N3}, & \text{if}\;k=0\;\text{and}\;\left(x={\hat{\alpha }}^{+}\left[k+2\right]\;\text{or}\;x={\hat{\alpha }}^{-}\left[k+2\right]\right)\\ & \left(\text{that}\;\text{is},\;\alpha \;\text{is}\;\text{null}\;\text{and}\;x\;\text{is}\;\text{the}\;3^{\prime }\;\text{cap}\;\text{in}\;\hat{\alpha }\right).\\ \text{T}, & \text{if}\;k\neq 0\;\text{and}\;\left(x={\hat{\alpha }}^{+}\left[2\right]\;\text{or}\;x={\hat{\alpha }}^{-}\left[2\right]\right)\\ & \left(\text{that}\;\text{is},\;\alpha \;\text{is}\;\text{not}\;\text{null}\;\text{and}\;x\;\text{is}\;\text{a}\;\text{tail}\;\text{in}\;\alpha \right).\\ \text{O}, & \text{otherwise}\text{.} \end{array}\right)$$

In addition, we define 5cap$\left(x,\;\hat{\alpha }\right)$ to be the 5' cap in the strand of $\hat{\alpha }$ that contains *x*. For convenience, we extend the definitions above from the capping contig to the capping genome. For instance, given a capping genome, say $\hat{\pi }$, char$\left(x,\;\hat{\pi }\right)$ denotes the character of *x *in a capping contig ${\hat{\pi }}_{i}$ of $\hat{\pi }$ that contains *x*, and 5cap$\left(x,\;\hat{\pi }\right)$ denotes the 5' cap of the strand in ${\hat{\pi }}_{i}$ containing *x*, that is, $\text{char}\left(x,\;\hat{\pi }\right)=\text{char}\left(x,\;{\hat{\pi }}_{i}\right)$ and 5cap$\left(x,\;\hat{\pi }\right)$ = 5cap$\left(x,\;{\hat{\pi }}_{i}\right)$. In our previous work [17], we have shown the following lemma.

**Lemma 4 (**[17]**) ***For a capping genome *$\hat{\pi }$*and *x∈Ê, *if *char $\left(x,\hat{\pi }\right)=C3$*(respectively*, *T)*, *then *$\text{char}\left(\hat{\pi }\hat{\text{Γ}}\left(x\right),\hat{\pi }\right)$*is T (respectively*, *C3) and if *$\text{char}\left(x,\hat{\pi }\right)=O$ (*respectively*, *N3 and C5)*, *then *$\text{char}\left(\hat{\pi }\hat{\text{Γ}}\left(x\right),\hat{\pi }\right)$*is O (respectively*, *N3 and C5)*.

Basically, we design our algorithm to solve the one-sided block ordering problem by dealing with the contigs of the capping genome $\hat{\pi }$ as if they were linear chromosomes. Let $c_{1}=\left(x,\;y\right)$ and $c_{2}=\left(u,\;v\right)$ be two 2-cycles with character pairs of (non-C5, non-C5) and (C5, C5), respectively, and let $c_{1}^{\prime }=\left(\hat{\pi }\hat{\text{Γ}}\left(y\right),\hat{\pi }\hat{\text{Γ}}\left(x\right)\right)$ and $c_{2}^{\prime }=\left(\hat{\pi }\hat{\text{Γ}}\left(v\right),\hat{\pi }\hat{\text{Γ}}\left(u\right)\right)$. Notice that the character pair of $c_{2}^{\prime }$ is (C5, C5) by Lemma 4. In our previous study [17], we have proven that performing a translocation  τ on $\hat{\pi }$ can be mimicked by the composition of $c_{2}^{\prime }c_{1}^{\prime }c_{2}c_{1}\hat{\pi }\left(\text{i}.\text{e}.,\;\tau =c_{2}^{\prime }c_{1}^{\prime }c_{2}c_{1}\right)$, if $\left(x,\;u\right)|\hat{\pi },\left(y,\;v\right)|\hat{\pi },\left(x,\;y\right)∤\hat{\pi }$ and $\left(x,\hat{\text{Γ}}\left(y\right)\right)∤\hat{\pi }$ (i.e., *x *and *u*, as well as *y *and *v*, lie in the same contig stand in $\hat{\pi }$, but *x *and *y *appear in the different contigs in $\hat{\pi }$). Moreover, if the character pair of $c_{1}$ is in CEpair = {(C3,C3),(C3,N3),(T,T),(T,N3),(N3,N3)}, then  τ acts on $\hat{\pi }$ by exchanging the two caps of some contig in $\hat{\pi }$ with the two caps of another contig and, as a result, leaves the original genome  π unaffected. Notice that the character pair of $c_{1}^{\prime }$ also belongs to CEpair and that of $c_{2}^{\prime }$ is (C5, C5) according to Lemma 4. Furthermore, if $c_{1}$ is a 2-cycle of character pair (T, C3) (respectively, (O, N3)), then performing τ on $\hat{\pi }$ becomes a fusion (respectively, fission) to act on π. Hence, we have the following lemma, where it can be verified that $\hat{\pi }\hat{\text{Γ}}\left(5\text{cap}\left(x,\hat{\pi }\right)\right)=5\text{cap}\left(\hat{\pi }\hat{\text{Γ}}\left(x\right),\hat{\pi }\right)$ and and $\hat{\pi }\hat{\text{Γ}}\left(5\text{cap}\left(y,\hat{\pi }\right)\right)=5\text{cap}\left(\hat{\pi }\hat{\text{Γ}}\left(y\right),\hat{\pi }\right)$.

**Lemma 5 (**[17]**) ***Let c*_1 _= (*x, y*) *denote a 2-cycle with *char$\left(x,\;\hat{\pi }\right)=T$*and *char$\left(y,\;\hat{\pi }\right)=C3$*, and let *$c_{2}=\left(5\text{cap}\left(x,\;\hat{\pi }\right),\;5\text{cap}\left(y,\hat{\pi }\right)\right)$, $c_{1}^{\prime }=\left(\hat{\pi }\hat{\text{Γ}}\left(y\right),\;\hat{\pi }\hat{\text{Γ}}\left(x\right)\right)$*and *$c_{2}^{\prime }=(\hat{\pi }\hat{\text{Γ}}\left(5\text{cap}\left(y,\;\hat{\pi }\right)\right)$, $\hat{\pi }\hat{\text{Γ}}\left(5\text{cap}\left(x,\;\hat{\pi }\right)\right))$. *If *$\left(x,\;y\right)∤\hat{\pi }$*and *$\left(x,\;\hat{\text{Γ}}\left(y\right)\right)\;∤\hat{\pi }$, *then the effect of *$c_{2}^{\prime }c_{1}^{\prime }c_{2}c_{1}\hat{\pi }$*is a fusion that acts on π by concatenating the contig containing y with the contig containing x*.

It is not hard to see that the permutation induced by an ordering of the uncapped genome *π *can be considered as the result of applying consecutive *m - *1 fusions to the *m *contigs in *π*. Based on the above discussion, it can be realized that our purpose is to find *m - *1 translocations to act on $\hat{\pi }$ such that their rearrangement effects on the original *π *are *m - *1 fusions and the genome rearrangement distance measured by weighted reversals and block-interchanges between the resulting assembly of the contigs in *π *and *σ *is minimum. In Algorithm 1 below, we describe our algorithm for efficiently solving the one-sided block ordering problem, where reversals are weighted one and block-interchanges are weighted two. Basically, we try to derive *m - *1 fusions from $\hat{\sigma }{\hat{\pi }}^{-1}$ to act on *π *in Algorithm 1.

Algorithm 1

**Input: **A partially assembled, linear, uni-chromosomal genome *π *= *π*_1_*π*_2 _... *π_m _*and a completely assembled, linear, uni-chromosomal genome *σ *= *σ*_1_.

**Output: **An optimally assembled genome of *π*, denoted by *assembly*(*π*), and the weighted reversal and block-interchange distance Δ(*π, σ*) between *assembly*(*π*) and *σ*.

**1: **Add *m - *1 null contigs $\sigma_{2},\sigma_{3},\ldots ,\sigma_{m}$ into *σ *such that $\sigma =\sigma_{1}\sigma_{2}\ldots \sigma_{m}$.

Obtain $\hat{\pi }={\hat{\pi }}_{1}{\hat{\pi }}_{2}\;\ldots \;{\hat{\pi }}_{m}$ and $\hat{\sigma }={\hat{\sigma }}_{1}{\hat{\sigma }}_{2}\;\ldots \;{\hat{\sigma }}_{m}$ by capping *π *and *σ*.

**2: **Compute $\hat{\sigma }{\hat{\pi }}^{-1}$ and $\hat{\pi }\hat{\text{Γ}}$.

3: /* To perform cap exchanges */

Let *i *= 0.

**while **there are *x *and *y *in a cycle of $\hat{\sigma }{\hat{\pi }}^{-1}$ such that $\left(\text{char}\left(x,\;\hat{\pi }\right),\;\text{char}\left(y,\;\hat{\pi }\right)\right)$ ∈ CEpair **do**

Let *i *= *i *+ 1.

Find *x *and *y *in a cycle of $\hat{\sigma }{\hat{\pi }}^{-1}$ with $\left(\text{char}\left(x,\;\hat{\pi }\right),\;\text{char}\left(y,\;\hat{\pi }\right)\right)$ ∈ CEpair.

Let $\chi_{i}=(\hat{\pi }\hat{\text{Γ}}\left(5\text{cap}\left(y,\;\hat{\pi }\right)\right)$, $\hat{\pi }\hat{\text{Γ}}\left(5\text{cap}\left(x,\;\hat{\pi }\right)\right))\;(\hat{\pi }\hat{\text{Γ}}\left(y\right)$, $\hat{\pi }\hat{\text{Γ}}\left(x)\right)\;(5\text{cap}\left(x,\;\hat{\pi }\right)$, $5\text{cap}\left(y,\;\hat{\pi }\right))\;\left(x,\;y\right)$.

Calculate new $\hat{\pi }=\chi_{i}\hat{\pi }$, new $\hat{\pi }\hat{\text{Γ}}=\chi_{i}\hat{\pi }\hat{\text{Γ}}$ and new $\hat{\sigma }{\hat{\pi }}^{-1}=\hat{\sigma }{\hat{\pi }}^{-1}\chi_{i}^{-1}$.

end while

4: /* To find consecutive m *- *1 fusions */

Let *i *= 0.

**while **there are two adjacent elements *x *and *y *in a cycle of $\hat{\sigma }{\hat{\pi }}^{-1}$ such that $\left(\text{char}\left(x,\;\hat{\pi }\right),\;\text{char}\left(y,\;\hat{\pi }\right)\right)=\left(\text{T},\;\text{C}3\right)$ and $\left(x,\;y\right)∤\hat{\pi }$**do**

Let *i *= *i *+ 1.

Find two adjacent elements *x *and *y *in a cycle of $\hat{\sigma }{\hat{\pi }}^{-1}$ such that $\left(\text{char}\left(x,\;\hat{\pi }\right),\;\text{char}\left(y,\;\hat{\pi }\right)\right)=\left(\text{T},\;\text{C}3\right)$ and $\left(x,\;y\right)∤\hat{\pi }$.

Let $\tau_{i}=(\hat{\pi }\hat{\text{Γ}}(5\text{cap}\left(y,\;\hat{\pi })\right)$, $\hat{\pi }\hat{\text{Γ}}\left(5\text{cap}\left(x,\;\hat{\pi }\right)\right))\;(\hat{\pi }\hat{\text{Γ}}\left(y\right)$, $\hat{\pi }\hat{\text{Γ}}\left(x\right))\;(5\text{cap}\left(x,\;\hat{\pi }\right)$, $5\text{cap}\left(y,\;\hat{\pi }\right))\;\left(x,\;y\right)$.

Calculate new $\hat{\pi }=\tau_{i}\hat{\pi }$, new $\hat{\pi }\hat{\text{Γ}}=\tau_{i}\hat{\pi }\hat{\text{Γ}}$ and new $\hat{\sigma }{\hat{\pi }}^{-1}=\hat{\sigma }{\hat{\pi }}^{-1}\tau_{i}^{-1}$.

end while

**while ***i < m - *1 **do**

Let *i *= *i *+ 1.

Find two adjacent elements *x *and *y *in a cycle of $\hat{\sigma }{\hat{\pi }}^{-1}$ such that $\left(\text{char}\left(x,\;\hat{\pi }\right),\;\text{char}\left(y,\;\hat{\pi }\right)\right)=\left(\text{T},\;\text{C}3\right)$ and $\left(x,\;y\right)|\hat{\pi }$.

Find the strand of a different contig in $\hat{\pi }$ with at least a non-cap integer and its 3' cap, say *z*, different from *y*.

Let $\tau_{i}=(\hat{\pi }\hat{\text{Γ}}\left(z\right)$, $\hat{\pi }\hat{\text{Γ}}\left(x\right))\;(\hat{\pi }\hat{\text{Γ}}\left(z\right)$, $\hat{\pi }\hat{\text{Γ}}\left(y)\right)\;\left(y,\;z\right)\left(x,\;z\right)$.

Calculate new $\hat{\pi }=\tau_{i}\hat{\pi }$, new $\hat{\pi }\hat{\text{Γ}}=\tau_{i}\hat{\pi }\hat{\text{Γ}}$ and new $\hat{\sigma }{\hat{\pi }}^{-1}=\hat{\sigma }{\hat{\pi }}^{-1}\tau_{i}^{-1}$.

end while

Let *assembly*(*π*) denote the assembled contig in current $\hat{\pi }$ whose caps are removed.

5: /* To find reversals */

Let $n_{\gamma }=0$.

**while **there are two adjacent elements *x *and *y *in a cycle of $\hat{\sigma }{\hat{\pi }}^{-1}$ such that $\left(x,\hat{\text{Γ}}\left(y\right)\right)|\hat{\pi }$**do**

Let $n_{\gamma }=n_{\gamma }+1$.

Find two adjacent elements *x *and *y *in a cycle of $\hat{\sigma }{\hat{\pi }}^{-1}$ such that $\left(x,\hat{\text{Γ}}\left(y\right)\right)|\hat{\pi }$.

Let $\gamma_{n_{\gamma }}=(\hat{\pi }\hat{\text{Γ}}\left(y\right)$, $\hat{\pi }\hat{\text{Γ}}\left(x\right))\left(x,\;y\right)$.

Calculate new $\hat{\pi }=\gamma_{n_{\gamma }}\hat{\pi }$, new $\hat{\pi }\hat{\text{Γ}}=\gamma_{n_{\gamma }}\hat{\pi }\hat{\text{Γ}}$ and new $\hat{\sigma }{\hat{\pi }}^{-1}=\hat{\sigma }{\hat{\pi }}^{-1}\gamma_{n_{\gamma }}^{-1}$.

end while

6: /* To find block-interchanges */

Let $n_{\beta }=0$.

while $\hat{\sigma }{\hat{\pi }}^{-1}\neq 1$ do

Let $n_{\beta }=n_{\beta }+1$.

Choose any two adjacent elements *x *and *y *in a cycle of $\hat{\sigma }{\hat{\pi }}^{-1}$.

Find two adjacent integers *u *and *v *in a cycle of $\hat{\sigma }{\hat{\pi }}^{-1}\left(x,\;y\right)$ such that $\left(u,\;v\right)∤\left(x,\;y\right)\hat{\pi }$.

Let $\beta_{\delta }=(\hat{\pi }\hat{\text{Γ}}\left(v\right)$, $\hat{\pi }\hat{\text{Γ}}\left(u\right))\;(\hat{\pi }\hat{\text{Γ}}\left(y\right)$, $\hat{\pi }\hat{\text{Γ}}\left(x\right))\;\left(u,\;v\right)\left(x,\;y\right)$.

Calculate new $\hat{\pi }=\beta_{n_{\beta }}\hat{\pi }$, new $\hat{\pi }\hat{\text{Γ}}=\gamma_{n_{\beta }}\hat{\pi }\hat{\text{Γ}}$ and new $\hat{\sigma }{\hat{\pi }}^{-1}=\hat{\sigma }{\hat{\pi }}^{-1}\beta_{n_{\beta }}^{-1}$.

end while

**7: **Output *assembly*(*π*) and $\text{Δ}\left(\pi ,\;\sigma \right)=n_{\gamma }+2n_{\beta }$.

Below, we consider an example to clarify Algorithm 1. Let *π *= {[1, 4], [-5, 6], [3, 2]} and *σ *= {[1, 2, ..., 6]} be the input linear, uni-chromosomal genomes of Algorithm 1. In our algorithm, these two genomes will be further represented by *π *= (1, 4)(-4, -1)(-5, 6)(-6, 5)(3, 2)(-2, -3) and *σ *= (1, 2, ..., 6)(-6*, -*5, ..., -1). First of all, we add two null contigs into *σ *and cap all the contigs in *π *and *σ *in a way such that $\hat{\pi }=\left(7,\;1,\;4,\;8\right)\;\left(-8,\;-4,\;-1,\;-7\right)\;\left(9,\;-5,\;6,\;10\right)\;\left(-10,\;-6,\;5,\;-9\right)\;\left(11,\;3,\;2,\;12\right)\left(-12,\;-2,\;-3,\;-11\right)$ and $\hat{\sigma }=\left(7,\;1,\;2,\;.\;.\;.\;,\;6,\;8\right)\;\left(-8,\;-6,\;-5,\;.\;.\;.\;,\;-1,\;-7\right)\;\left(9,\;10\right)\;\left(-10,\;-9\right)\;\left(11,\;12\right)\;\left(-12,\;-11\right)$. Next, we compute $\hat{\sigma }{\hat{\pi }}^{-1}=\left(2,\;4\right)\;\left(-1,\;-3\right)\;\left(3,\;12\right)\;\left(-2,\;-11\right)\;\left(5,\;-5,\;10,\;8\right)\;\left(-4,\;-6,\;-9,\;6\right)$. It can be found that 10 and 8 are in a cycle of current $\hat{\sigma }{\hat{\pi }}^{-1}$ with $\left(\text{char}\;\left(10,\;\hat{\pi }\right),\;\text{char}\left(8,\;\hat{\pi }\right)\right)=\left(\text{C}3,\;\text{C}3\right)\in \text{CEpair}$. We perform a cap exchange on $\hat{\pi }$ by multiplying $(\hat{\pi }\hat{\text{Γ}}(5\text{cap}\left(8,\;\hat{\pi })\right)$, $(\hat{\pi }\hat{\text{Γ}}\left(5\text{cap}\left(8,\;\hat{\pi }\right)\right)$, $\;\hat{\pi }\hat{\text{Γ}}\left(5\text{cap}\left(10,\;\hat{\pi }\right)\right))\;(\hat{\pi }\hat{\text{Γ}}\left(8\right)$, $\hat{\pi }\hat{\text{Γ}}\left(10\right))\;(5\text{cap}\left(10,\hat{\pi }\right)$$\;5\text{cap}\left(8,\;\hat{\pi }\right))\;\left(10,\;8\right)=\left(-8,\;-10\right)\;\left(-4,\;-6\right)\;\left(9,7\right)\;\left(10,8\right)$ with $\hat{\pi }$, resulting in new $\hat{\pi }=\left(7,\;1,\;4,\;10\right)\;\left(-10,\;-4,\;-1,\;-7\right)\;\left(9,\;-5,\;6,\;8\right)\;\left(-8,\;-6,\;5,\;-9\right)\;\left(11,\;3,\;2,\;12\right)\;\left(-12,\;-2,\;-3,\;-11\right)$. In addition, we have new $\hat{\sigma }{\hat{\pi }}^{-1}=\left(2,\;4\right)\left(-1,\;-3\right)\;\left(3,\;12\right)\;\left(-2,\;-11\right)\;\left(5,\;-5,\;10\right)\;\left(-4,\;-9,\;6\right)\;\left(9,\;7\right)\;\left(-10,\;-8\right)$. It can be observed that -5 and 10 are in the same cycle of $\hat{\sigma }{\hat{\pi }}^{-1}$ with satisfying that $\text{char}\left(--5,\;\hat{\pi }\right)=\text{T}$, $\text{char}\left(10,\;\hat{\pi }\right)=\text{C}3$ and $\left(-5,\;10\right)∤\hat{\pi }$ (since -5 and 10 are in different contigs in current $\hat{\pi }$). Therefore, we perform a fusion on $\hat{\pi }$, by multiplying $\rho_{1}=(\hat{\pi }\hat{\text{Γ}}\left(5\text{cap}\left(10,\;\hat{\pi }\right)\right)$, $\hat{\pi }\hat{\text{Γ}}\left(5\text{cap}\left(-5,\;\hat{\pi }\right)\right))\;(\hat{\pi }\hat{\text{Γ}}\left(10\right)$, $\hat{\pi }\hat{\text{Γ}}\left(-5\right))\;(5\text{cap}\left(-5,\;\hat{\pi }\right)$, $5\text{cap}\left(10,\;\hat{\pi }\right))\;\left(-5,\;10\right)=$(-10,-8)(-4,-9)(9,7)(-5,10) with $\hat{\pi }$, to obtain new $\hat{\pi }=\left(7,\;1,\;4,\;-5,\;6,\;8\right)\;\left(-8,\;-6,\;5,\;-4,\;-1,\;-7\right)\;\left(9,10\right)\;\left(-10,\;-9\right)\;\left(11,\;3,\;2,\;12\right)\;\left(-12,\;-2,\;-3\;-11\right)$. Moreover, we have new $\hat{\sigma }{\hat{\pi }}^{-1}=\left(2,\;4\right)\;\left(-1,\;-3\right)\;\left(3,\;12\right)\;\left(-2,\;-11\right)\;\left(5,\;-5\right)\;\left(-4,\;6\right)$, in which 3 and 12 form a (T, C3) pair but they belong to the same contig strand in $\hat{\pi }$, that is, $\left(3,\;12\right)|\hat{\pi }$. In this case, $\hat{\pi }$ has a contig strand (7, 1, 4, -5, 6, 8) whose 3' cap is 8 that is different from 12. Hence, we multiply $(\hat{\pi }\hat{\text{Γ}}\left(8\right)$, $\hat{\pi }\hat{\text{Γ}}\left(3\right))\;(\hat{\pi }\hat{\text{Γ}}\left(8\right)$, $\hat{\pi }\hat{\text{Γ}}\left(12\right))\;\left(12,\;8\right)\left(3,\;8\right)=\left(-6,\;-11\right)\;\left(-6,\;-2\right)\;\left(12,\;8\right)\;\left(3,\;8\right)$ with $\hat{\pi }$ to obtain new $\hat{\pi }=\left(7,\;1,\;4,\;-5,\;6,\;3,\;2,\;8\right)\;\left(-8,\;-2,\;-3,\;-6,\;5,\;-4,\;-1,\;-7\right)\;\left(9,\;10\right)\;\left(-10,\;-9\right)\;\left(11,\;12\right)\;\left(-12,\;-11\right)$ and new $\hat{\sigma }{\hat{\pi }}^{-1}=\left(2,\;4\right)\;\left(-1,\;-3\right)\;\left(5,\;-5\right)\;\left(-4,\;6\right)\;\left(3,\;8\right)\;\left(-2,\;-6\right)$. Notice that -4 and 6 are adjacent in a cycle of current $\hat{\sigma }{\hat{\pi }}^{-1}$ and they are in different strands in current $\hat{\pi }$ since $\left(-4,\;\hat{\text{Γ}}\left(6\right)\right)|\hat{\pi }$. Thus, we can find a reversal, which is $\left(\hat{\pi }\hat{\text{Γ}}\left(6\right),\;\hat{\pi }\hat{\text{Γ}}\left(-4\right)\right)\left(-4,\;6\right)=\left(5,\;-5\right)\;\left(-4,6\right)$, from $\hat{\sigma }{\hat{\pi }}^{-1}$ to transform $\hat{\pi }$ into (7, 1, 4, 5, 6, 3, 2, 8) (-8, -2, -3, -6, -5, -4, -1, -7) (9, 10) (-10, -9) (11, 12) (-12, -11). After that, we have new $\hat{\sigma }{\hat{\pi }}^{-1}=\left(2,\;4\right)\;\left(-1,\;-3\right)\;\left(3,\;8\right)\;\left(-2,\;-6\right)$, which can serve as a block-interchange to further transform $\hat{\pi }$ into (7, 1, 2, 3, 4, 5, 6, 8)(-8, -6, -5, -4, -3, -2, -1, -7) (9, 10) (-10, -9) (11, 12) (-12, -11), which is equal to $\hat{\sigma }$. As a result, we obtain an ordering ([1,4], [-5, 6], [3,2]) of *π *whose induced permutation [1,4] ⊙ [-5, 6] ⊙ [3,2] = (1, 4, -5, 6, 3, 2) can be transformed into the permutation (1, 2, ..., 6) of *σ *using a reversal and a block-interchange (i.e., Δ(*π, σ*) = 3).

Actually, after running the step 3 of Algorithm 1, it can be verified according to the capping of *π *and *σ *and Lemma 3 that for any two adjacent elements *x *and *y *in a cycle of $\hat{\pi }{\hat{\sigma }}^{-1}$ with $(\text{char}\left(x,\;\hat{\pi }\right)$, $\text{char}\left(y,\;\hat{\pi }\right))=\left(\text{T},\;\text{C}3\right)$, if $\left(x,\;y\right)∤\hat{\pi }$, then $\left(x,\hat{\text{Γ}}\left(y\right)\right)∤\hat{\pi }$. Moreover, the operation $\tau_{i}=\left(\hat{\pi }\hat{\text{Γ}}\left(z\right),\;\hat{\pi }\hat{\text{Γ}}\left(x\right)\right)\;\left(\hat{\pi }\hat{\text{Γ}}\left(z\right),\;\hat{\pi }\hat{\text{Γ}}\left(y\right)\right)\;\left(y,\;z\right)\left(x,\;z\right)$ used in the step 4 of Algorithm 1 acts on $\hat{\pi }$ still as a fusion of *π*, as explained as follows. Notice that $\left(x,\;y\right)|\hat{\pi }$, meaning that *x *and *y *are in the same cycle of $\hat{\pi }$ and hence $5\text{cap}\left(x,\;\hat{\pi }\right)=5\text{cap}\left(y,\;\hat{\pi }\right)$. It can be verified that $(5\text{cap}\left(y,\;\hat{\pi }\right)$, $5\text{cap}\left(z,\;\hat{\pi }\right))\;(5\text{cap}\left(x,\;\hat{\pi }\right)$, $5\text{cap}\left(z,\;\hat{\pi }\right))=1$. Since $\left(x,\;y\right)|\hat{\pi }$, we have $(\hat{\pi }\hat{\text{Γ}}\left(y\right),\;\hat{\pi }\hat{\text{Γ}}\left(x\right))|\hat{\pi }$ and hence $(5\text{cap}\left(\hat{\pi }\hat{\text{Γ}}\left(z\right),\;\hat{\pi }\right)$, $5\text{cap}\left(\hat{\pi }\hat{\text{Γ}}\left(y\right),\;\hat{\pi }\right))(5\text{cap}\left(\hat{\pi }\hat{\text{Γ}}\left(z\right),\;\hat{\pi }\right)$, $5\text{cap}\left(\hat{\pi }\hat{\text{Γ}}\left(x\right),\hat{\pi }\right))=1$. It is not hard to see that $(\hat{\pi }\hat{\text{Γ}}\left(z\right),\;\hat{\pi }\hat{\text{Γ}}\left(x\right))(\hat{\pi }\hat{\text{Γ}}\left(z\right),\;\hat{\pi }\hat{\text{Γ}}\left(y\right))=(\hat{\pi }\hat{\text{Γ}}\left(x\right),\;\hat{\pi }\hat{\text{Γ}}\left(y\right))(\hat{\pi }\hat{\text{Γ}}\left(z\right),\;\hat{\pi }\hat{\text{Γ}}\left(x\right))$. Thus, τ*_i _*can be rewritten as $\tau_{i}=\alpha_{2}\alpha_{1}$, where $\alpha_{1}=(5\text{cap}\left(\hat{\pi }\hat{\text{Γ}}\left(z\right),\;\hat{\pi }\right)$, $5\text{cap}\left(\hat{\pi }\hat{\text{Γ}}\left(x\right),\;\hat{\pi }\right))(\hat{\pi }\hat{\text{Γ}}\left(z\right),\;\hat{\pi }\hat{\text{Γ}}\left(x\right))(5\text{cap}\left(x,\;\hat{\pi }\right)$, $5\text{cap}\left(z,\;\hat{\pi }\right))\left(x,\;z\right)$ and $\alpha_{2}=(5\text{cap}\left(\hat{\pi }\hat{\text{Γ}}\left(z\right),\;\hat{\pi }\right)$, $5\text{cap}\left(\hat{\pi }\hat{\text{Γ}}\left(y\right),\;\hat{\pi }\right))(\hat{\pi }\hat{\text{Γ}}\left(x\right),\hat{\pi }\hat{\text{Γ}}\left(y\right))(5\text{cap}\left(y,\;\hat{\pi }\right)$, $5\text{cap}\left(z,\;\hat{\pi }\right))\left(y,\;z\right)$. It can be verified that $\alpha_{2}=(5\text{cap}\left(\alpha_{1}\hat{\pi }\hat{\text{Γ}}\left(z\right),\;\alpha_{1}\hat{\pi }\right)$, $5\text{cap}\left(\alpha_{1}\hat{\pi }\hat{\text{Γ}}\left(y\right),\;\alpha_{1}\hat{\pi }\right))(\alpha_{1}\hat{\pi }\hat{\text{Γ}}\left(z\right),\;\alpha_{1}\hat{\pi }\hat{\text{Γ}}\left(y\right))(5\text{cap}\left(z,\;\alpha_{1}\hat{\pi }\right)$, $5\text{cap}\left(y,\;\alpha_{1}\hat{\pi }\right))\left(y,\;z\right)$. By Lemma 5, as well as the previous discussion, it can be realized that *a*_1 _acts on $\hat{\pi }$ as a fusion of *π *and *α*_2 _continues to act on $\alpha_{1}\hat{\pi }$ as a cap exchange. As a result, the rearrangement effect of acting *τ_i _*on $\hat{\pi }$ is still equivalent to a fusion acting on *π*. The above discussion indicates that a fusion to *π *can be mimicked by a translocation *τ*, which acts on $\hat{\pi }$ as a fusion of *π*, followed by zero or more translocations acting on $\tau \hat{\pi }$ as cap exchanges.

In the following, we prove the correctness of Algorithm 1. Initially, it is not hard to see that all the 5' caps are fixed in $\hat{\sigma }{\hat{\pi }}^{-1}$ and $\text{char}\left(x,\;\hat{\pi }\right)\neq \text{N}3$ for all $x\in \hat{E}$. For any element $x\in \hat{E}$ with $\text{char}\left(x,\;{\hat{\pi }}_{i}\right)=\text{T}$, where $1\leq i\leq m,\;\text{if}\;{\hat{\pi }}^{-1}\left(x\right)\neq {\hat{\sigma }}_{1}^{+}\left[1\right]$ and ${\hat{\pi }}^{-1}\left(x\right)\neq {\hat{\sigma }}_{1}^{-}\left[1\right]$, that is, the 5' cap of ${\hat{\pi }}_{i}$ is not equal to that of ${\hat{\sigma }}_{1}$, then the character of $\hat{\sigma }{\hat{\pi }}^{-1}\left(x\right)$ in $\hat{\pi }$ must be C3. If any cycle in $\hat{\sigma }{\hat{\pi }}^{-1}$ contains any two elements *x *and *y *with the same character (either T or C3) in $\hat{\pi }$, then we can extract two 2-cycles *c*_1 _= (*x, y*) and $c_{1}^{\prime }=(\hat{\pi }\hat{\text{Γ}}\left(y\right),\;\hat{\pi }\hat{\text{Γ}}\left(x\right))$ from two mate cycles in $\hat{\sigma }{\hat{\pi }}^{-1}$ and multiply $c_{2}^{\prime }c_{1}^{\prime }c_{2}c_{1}$ with $\hat{\pi }$ to exchange the caps of the contigs containing *x *and *y*, respectively, in $\hat{\pi }$, where $c_{2}=(5\text{cap}\left(x,\;\hat{\pi }\right),\;5\text{cap}\left(y,\;\hat{\pi }\right))$ and $c_{2^{\prime }}=(\hat{\pi }\hat{\text{Γ}}\left(5\text{cap}\left(y,\hat{\pi }\right)),\hat{\pi }\hat{\text{Γ}}(5\text{cap}\left(x,\hat{\pi }\right))\right)$. This is the job to be performed in the step 3 in Algorithm 1. Moreover, after finishing the cap exchanges in the step 3, each cycle in the remaining $\hat{\sigma }{\hat{\pi }}^{-1}$ has at most one element with T character and at most one element with C3 character. In other words, after running the step 3, there are at least 2(*m-*1) cycles in the resulting $\hat{\sigma }{\hat{\pi }}^{-1}$ such that each such a cycle contains exactly one element, say *x*, with $\left(x,\hat{\pi }\right)=\text{T}$ and exactly one element, say *y*, with $\text{char}\left(y,\;\hat{\pi }\right)=\text{C}3$, and $\hat{\sigma }{\hat{\pi }}^{-1}\left(x\right)=y$. In this case, we can further derive 2(*m - *1) 2-cycles from these cycles in $\hat{\sigma }{\hat{\pi }}^{-1}$ with each 2-cycle having a character pair of (T, C3). Intriguingly, we shall show below that these 2(*m-*1) 2-cycles with character pair (T, C3), denoted by $f_{1},f_{1}^{\prime },.\;.\;.\;,\;f_{m-1},f_{m-1}^{\prime }$, can be used to obtain an optimal ordering of *π *such that the weighted reversal and block-interchange distance between the permutation induced by this ordering of *π *and *σ *is minimum. In fact, *f_k _*and $f_{k}^{\prime }$, where 1 ≤ *k *≤ *m - *1, are derived from two mate cycles in $\hat{\sigma }{\hat{\pi }}^{-1}$ and hence we call them as *mate *2-cycles below. Moreover, if $f_{k}=\left(x,\;y\right)$, then $f_{k}^{\prime }=(\hat{\pi }\hat{\text{Γ}}\left(y\right),\;\hat{\pi }\hat{\text{Γ}}\left(x\right))$.

For 1 ≤ *k *≤ *m - *1, we simply let $f_{k}=\left(x_{k},\;y_{k}\right)$, where $\text{char}\left(x_{k},\;\hat{\pi }\right)=\text{T}$ and $\text{char}\left(y_{k},\;\hat{\pi }\right)=\text{C}3$. Then $f_{k}^{\prime }=(\hat{\pi }\hat{\text{Γ}}\left(y_{k}\right),\;\hat{\pi }\hat{\text{Γ}}\left(x_{k}\right))$. As mentioned previously, the permutation induced by an ordering of *π *can be mimicked by performing *m - *1 consecutive fusions on *π *that has *m *contigs initially. According to Lemma 5 and our previous discussion, if $f_{k}∤\hat{\pi }$, where 1 ≤ *k *≤ *m - *1, then $g_{k}^{\prime }f_{k}^{\prime }g_{k}f_{k}$ can be applied to $\hat{\pi }$ to function as a fusion of two contigs in *π*, where $g_{k}=(5\text{cap}\left(x_{k},\;\hat{\pi }\right),\;5\text{cap}\left(y_{k},\;\hat{\pi }\right))$ and $g_{k}^{\prime }=(\hat{\pi }\hat{\text{Γ}}\left(5\text{cap}\left(y,\;\hat{\pi }\right)),\;\hat{\pi }\hat{\text{Γ}}(5\text{cap}\left(x,\;\hat{\pi }\right))\right)$. Notice that *g_k _*and $g_{k}^{\prime }$ are mate 2-cycles. However, not all $f_{1},f_{2},\ldots ,f_{m-1}$ cannot divide $\hat{\pi }$. Suppose that only the first *λ *2-cycles $f_{1},f_{2},\ldots ,f_{\lambda }$ cannot divide $\hat{\pi }$, where 0 ≤ λ ≤ *m *- 1, that is, $f_{k}∤\hat{\pi }$ for 1 ≤ *k *≤ *λ*, but $f_{k}|\hat{\pi }$ for λ + 1 ≤ *k *≤ *m *- 1. In this situation, we shall show below that we still can use $f_{1},f_{2},\ldots ,f_{m-1}$, as well as their mate 2-cycles, to derive an optimal ordering of *π*, as we did in the step 4 in Algorithm 1.

Recall that the 5' caps are all fixed in the beginning $\hat{\sigma }{\hat{\pi }}^{-1}$ (before the step 3 in Algorithm 1). As mentioned before, for any translocation used to perform on $\hat{\pi }$, it can be expressed as four 2-cycles, two with (non-C5, non-C5) character pair and the others with (C5, C5). It can be verified that during the process of the step 3, no two elements *x *and *y *with char $\left(x,\hat{\pi }\right)$ = C5 but char $\left(y,\hat{\pi }\right)\neq \text{C}5$ can be found in a cycle of the $\hat{\sigma }{\hat{\pi }}^{-1}$[17], that is, C5 and non-C5 elements are not mixed together in the same cycle of $\hat{\sigma }{\hat{\pi }}^{-1}$. Actually, this property still continues to be asserted when we later perform any translocation on $\hat{\pi }$ to function as a fusion of *π*. Let us now pay attention on those cycles in $\hat{\sigma }{\hat{\pi }}^{-1}$ with only non-C5 elements and temporarily denote the composition of these cycles by $\phi \left(\hat{\sigma }{\hat{\pi }}^{-1}\right)$. If we still can find any two elements *x *and *y *from a cycle in $\phi \left(\hat{\sigma }{\hat{\pi }}^{-1}\right)$ such that $(\hat{\pi }\hat{\text{Γ}}(5\text{cap}\left(y,\;\hat{\pi }\right))$, $\hat{\pi }\hat{\text{Γ}}(5\text{cap}\left(x,\;\hat{\pi }\right)))(\hat{\pi }\hat{\text{Γ}}\left(y\right),\;\hat{\pi }\hat{\text{Γ}}\left(x\right))(5\text{cap}\left(x,\;\hat{\pi }\right)$, $5\text{cap}\left(y,\;\hat{\pi }\right))\left(x,\;y\right)$ is an exchange of caps when applying it to $\hat{\pi }$, then we apply this cap exchange to $\hat{\pi }$ until we cannot find any one from $\phi \left(\hat{\sigma }{\hat{\pi }}^{-1}\right)$. Finally, we denote such a $\phi \left(\hat{\sigma }{\hat{\pi }}^{-1}\right)$ without any cap exchange by $\psi \left(\hat{\sigma }{\hat{\pi }}^{-1}\right)$. Basically, $\psi \left(\hat{\sigma }{\hat{\pi }}^{-1}\right)$ can be considered as a permutation of $E^{\prime }=E\cup \left\{-c_{2i},\;c_{2i+1}\;:\;0\leq i\leq m-1\right\}$ and hence its norm $\left||\psi \left(\hat{\sigma }{\hat{\pi }}^{-1}\right)|\right|$ is equal to $|E^{\prime }|-\;n_{c}(\psi \left(\hat{\sigma }{\hat{\pi }}^{-1}\right))$ according to the formula we mentioned before.

**Lemma 6 **Let $\tau =(\hat{\pi }\hat{\text{Γ}}(5\text{cap}\left(y,\;\hat{\pi }\right))$, $\hat{\pi }\hat{\text{Γ}}(5\text{cap}\left(x,\;\hat{\pi }\right)))(\hat{\pi }\hat{\text{Γ}}\left(y\right),\;\hat{\pi }\hat{\text{Γ}}\left(x\right))(5\text{cap}\left(x,\;\hat{\pi }\right)$, $5\text{cap}\left(y,\hat{\pi }\right))\left(x,\;y\right)$*be a fusion to act on π*, *where *char$\left(x,\hat{\pi }\right)=T$*and *char$\left(y,\hat{\pi }\right)=C3$. *Then *$||\psi \left(\hat{\sigma }{\hat{\pi }}^{-1}\right)||-||\psi \left(\hat{\sigma }{\hat{\pi }}^{-1}\tau^{-1}\right)||\;\in \left\{-2,0,2\right\}$.

*Proof*. For simplicity, it is assumed that we cannot find any cap exchange from $\hat{\sigma }{\hat{\pi }}^{-1}$ to perform on $\hat{\pi }$. We then consider the following two cases.

Case 1: Suppose that $\left(x,\;y\right)|\hat{\sigma }{\hat{\pi }}^{-1}$, that is, both *x *and *y *lie in the same cycle, say *α*, in $\hat{\sigma }{\hat{\pi }}^{-1}$. Without loss of generality, let $\alpha =\left(a_{1},\;a_{2},\;\ldots ,\;a_{i}\equiv x,\;\ldots ,\;a_{j}\equiv \;y\right)$. Then *α *can be expressed as *α *= *α*_1_*α*_2_(*x*, *y*), where *α*_1 _= (*a*_1_, ..., *a_i_*}) and *α*_2 _= (a_*i*+1_, ..., *a_j_*). Let $\alpha^{\prime }$ denote the mate cycle of *α *in $\hat{\sigma }{\hat{\pi }}^{-1}$, that is, $\alpha^{\prime }=(\hat{\pi }\hat{\text{Γ}}\left(a_{j}\right),\;\ldots ,\hat{\pi }\hat{\text{Γ}}\left(a_{i}\right),\;\ldots ,\hat{\pi }\hat{\text{Γ}}\left(a_{2}\right),\hat{\pi }\hat{\text{Γ}}\left(a_{1}\right))$. Then it can be expressed as $\alpha^{\prime }=\alpha_{1}^{\prime }\alpha_{2}^{\prime }(\hat{\pi }\hat{\text{Γ}}\left(y\right),\;\hat{\pi }\hat{\text{Γ}}\left(x\right))$, where $\alpha_{1}^{\prime }=(\hat{\pi }\hat{\text{Γ}}\left(a_{i-1}\right),\;\ldots ,\hat{\pi }\hat{\text{Γ}}\left(a_{1}\right),\;\hat{\pi }\hat{\text{Γ}}\left(a_{j}\right))$ and $\alpha_{2}^{\prime }=(\hat{\pi }\hat{\text{Γ}}\left(a_{j-1}\right),\;\hat{\pi }\hat{\text{Γ}}\left(a_{j-2}\right),\;\ldots ,\hat{\pi }\hat{\text{Γ}}\left(a_{i}\right))$. Clearly, after applying *τ *to $\hat{\pi }$, the cycle *α *becomes two disjoint cycles *α*_1 _and *α*_2 _in $\hat{\sigma }{\hat{\pi }}^{-1}\tau^{-1}$ and $\alpha^{\prime }$ becomes two disjoint $\alpha_{1}^{\prime }$ and $\alpha_{2}^{\prime }$. It means that $n_{c}(\psi \left(\hat{\sigma }{\hat{\pi }}^{-1}\tau^{-1}\right))=n_{c}(\psi \left(\hat{\sigma }{\hat{\pi }}^{-1}\right))+2$ and hence $||\psi \left(\hat{\sigma }{\hat{\pi }}^{-1}\right)||-||\psi \left(\hat{\sigma }{\hat{\pi }}^{-1}\tau^{-1}\right)||\;=2$.

Case 2: Suppose that $\left(x,\;y\right)∤\hat{\sigma }{\hat{\pi }}^{-1}$, that is, *x *and *y *lie in two different cycles, say *α*_1 _and *α*_2_, in $\hat{\sigma }{\hat{\pi }}^{-1}$. In this case, $\hat{\pi }\hat{\text{Γ}}\left(x\right)$ and $\hat{\pi }\hat{\text{Γ}}\left(y\right)$ also are in two different cycles, say $\alpha_{1}^{\prime }$ and $\alpha_{2}^{\prime }$, that are the mate cycles of *α*_1 _and *α*_2_, respectively, in $\hat{\sigma }{\hat{\pi }}^{-1}$. By Lemma 4, char $\left(\hat{\pi }\hat{\text{Γ}}\left(x\right),\hat{\pi }\right)=\text{C}3$ and char $\left(\hat{\pi }\hat{\text{Γ}}\left(y\right),\hat{\pi }\right)=\text{T}$. Then performing *τ *on $\hat{\pi }$ leads *α*_1 _and *α*_2 _to be joined together into a cycle, say *α*, in $\hat{\sigma }{\hat{\pi }}^{-1}\tau^{-1}$ and $\alpha_{1}^{\prime }$ and $\alpha_{2}^{\prime }$ to be joined into another cycle, say $\alpha^{\prime }$. If *α*_1 _and *α*_2_, as well as $\alpha_{1}^{\prime }$ and $\alpha_{2}^{\prime }$, does not contain both T and C3 elements simultaneously, then $n_{c}(\psi \left(\hat{\sigma }{\hat{\pi }}^{-1}\tau^{-1}\right))=n_{c}(\psi \left(\hat{\sigma }{\hat{\pi }}^{-1}\right))-2$ and hence $||\psi \left(\hat{\sigma }{\hat{\pi }}^{-1}\right)||-||\psi \left(\hat{\sigma }{\hat{\pi }}^{-1}\tau^{-1}\right)||\;=-2$. If exactly one of *α*_1 _and *α*_2_, as well as exactly one of $\alpha_{1}^{\prime }$ and $\alpha_{2}^{\prime }$, contains both T and C3 elements simultaneously, then joining *α*_1 _and *α*_2 _will also change char $\left(x,\;\hat{\pi }\right)$ from T to O and char $\left(y,\;\hat{\pi }\right)$ from C3 to N3, and joining $\alpha_{1}^{\prime }$ and $\alpha_{2}^{\prime }$ will change char $\left(\hat{\pi }\hat{\text{Γ}}\left(x\right),\;\hat{\pi }\right)$ from C3 to N3 and char $\left(\hat{\pi }\hat{\text{Γ}}\left(y\right),\;\hat{\pi }\right)$ from T to O. Therefore, the cycle *α*, as well as $\alpha^{\prime }$, contains a C3 (or T) element and an N3 element. In this case, we can use these four elements, along with their corresponding 5' caps in $\hat{\pi }$, as a cap exchange to perform on $\hat{\pi }$, resulting in that each of the cycles *α *and $\alpha^{\prime }$ is divided into two smaller ones in new $\hat{\sigma }{\hat{\pi }}^{-1}$. As a result, $n_{c}(\psi \left(\hat{\sigma }{\hat{\pi }}^{-1}\tau^{-1}\right))=n_{c}(\psi \left(\hat{\sigma }{\hat{\pi }}^{-1}\right))$ and hence $||\psi \left(\hat{\sigma }{\hat{\pi }}^{-1}\right)||-||\psi \left(\hat{\sigma }{\hat{\pi }}^{-1}\tau^{-1}\right)||\;=0$. Suppose that both *α*_1 _and *α*_2_, as well as both $\alpha_{1}^{\prime }$ and $\alpha_{2}^{\prime }$, contain T and C3 elements at the same time. Then, after applying *τ *to $\hat{\pi }$, one of the above two T elements becomes an O element in new $\hat{\pi }$, leading to *α*, as well as $\alpha^{\prime }$, containing only a T element, along with a C3 element and an N3 element. Next, we can use the T and N3 elements (or the C3 and N3 elements) in *α *and $\alpha^{\prime }$ and their corresponding 5' caps in $\hat{\pi }$ to exchange the caps of $\hat{\pi }$. After that, *α*, as well as $\alpha^{\prime }$, is divided into two cycles in the new $\hat{\sigma }{\hat{\pi }}^{-1}$ and, consequently, $n_{c}(\psi \left(\hat{\sigma }{\hat{\pi }}^{-1}\tau^{-1}\right))=n_{c}(\psi \left(\hat{\sigma }{\hat{\pi }}^{-1}\right))$ and hence $||\psi \left(\hat{\sigma }{\hat{\pi }}^{-1}\right)||-||\psi \left(\hat{\sigma }{\hat{\pi }}^{-1}\tau^{-1}\right)||\;=0$.

Notice that if $\hat{\pi }=\hat{\sigma }$, then $||\psi \left(\hat{\sigma }{\hat{\pi }}^{-1}\right)||\;=0$. According to Lemmas 5 and 6, any translocation *τ *that acts on $\hat{\pi }$ as a fusion of *π *decreases the norm $\left||\psi \left(\hat{\sigma }{\hat{\pi }}^{-1}\right)|\right|$ at most by two. Hence, we call *τ *as a *good *fusion of *π *if $||\psi \left(\hat{\sigma }{\hat{\pi }}^{-1}\right)||-||\psi \left(\hat{\sigma }{\hat{\pi }}^{-1}\tau^{-1}\right)||\;=2$. By the discussion in the proof of Lemma 6, we have the following corollary.

**Corollary 1 ***Let *$\tau =(\hat{\pi }\hat{\text{Γ}}(5\text{cap}\left(y,\;\hat{\pi }\right))$, $\hat{\pi }\hat{\text{Γ}}(5\text{cap}\left(x,\;\hat{\pi }\right)))(\hat{\pi }\hat{\text{Γ}}\left(y\right)$, $\hat{\pi }\hat{\text{Γ}}\left(x\right))(5\text{cap}\left(x,\;\hat{\pi }\right)$, $5\text{cap}\left(y,\;\hat{\pi }\right))\left(x,\;y\right)$*be a fusion to act on π, where *char $\left(x,\hat{\pi }\right)=T$*and *char $\left(y,\hat{\pi }\right)=C3$. *If *$\left(x,\;y\right)|\hat{\sigma }{\hat{\pi }}^{-1}$, *then *τ *is a good fusion to perform on π*.

According to Corollary 1, it can be realized that *f_k_*, as well as its mate 2-cycle $f_{k}^{\prime }$, can derive a good fusion to act on *π*, where 1 ≤ *k *≤ *λ*. If *λ *= *m *- 1, then performing the *m *- 1 fusions on *π*, as we did in Algorithm 1, corresponds to an optimal ordering of *π *such that the weighted reversal and block-interchange distance between the assembly of *π *and *σ *is minimum. For simplifying our discussion below, we assume that the λ good fusions derived from *f*_1_, *f*_2_, ..., *f*_λ _and their mate 2-cycles can assemble λ + 1 contigs of *π *into several super-contigs. If λ <*m *- 1, then we show below that the fusions of *m *- 1 contigs in *π *performed by our algorithm utilizing *f*_1_, *f*_2_, ..., *f*_*m*-1 _is still optimal.

**Lemma 7 ***Let *$\tau_{1},\tau_{2},\ldots ,\tau_{m-1}$*be any sequence of m *- 1 *translocations that act on *$\hat{\pi }$*as fusions to assemble m *- 1 *contigs in π*. *Let *${\hat{\omega }}_{k}$*be the genome obtained by performing τ_k _and zero or more following cap exchanges on *${\hat{\omega }}_{k-1}$*such that no more cap exchange can be derived from *$\hat{\sigma }{\hat{\omega }}_{k}^{-1}$, *where *${\hat{\omega }}_{0}=\hat{\pi }$*and *1 ≤ *k *≤ *m *- 1. *Then *$||\psi \left(\hat{\sigma }{\hat{\omega }}_{0}^{-1}\right)||-||\psi \left(\hat{\sigma }{\hat{\omega }}_{m-1}^{-1}\right)||\;\leq 2\lambda$.

*Proof*. For simplicity, we assume that in the beginning, no cap exchange can be derived from $\hat{\sigma }{\hat{\omega }}_{0}^{-1}$ to act on ${\hat{\omega }}_{0}$. Let $\omega_{k}$ denote the genome obtained from ${\hat{\omega }}_{k}$ by removing its caps, where 1 ≤ *k *≤ *m *- 1. By Lemma 6, $||\psi \left(\hat{\sigma }{\hat{\omega }}_{k-1}^{-1}\right)||-||\psi \left(\hat{\sigma }{\hat{\omega }}_{k}^{-1}\right)||\;\in \left\{-2,0,2\right\}$ and by Corollary 1, $||\psi \left(\hat{\sigma }{\hat{\omega }}_{k-1}^{-1}\right)||-||\psi \left(\hat{\sigma }{\hat{\omega }}_{k}^{-1}\right)||\;=2$ if $\tau_{k}$ is a good fusion to $\omega_{k-1}$. In fact, there are at most *λ *translocations from $\tau_{1},\tau_{2},\ldots ,\tau_{m-1}$ that are good fusions. The reason is as follows. As mentioned before, we can obtain 2λ 2-cycles $f_{1},f_{1}^{\prime },\ldots ,f_{\lambda },f_{\lambda }^{\prime }$ from $\hat{\sigma }{\hat{\pi }}^{-1}$ that can derive λ good fusions to act on *π*, say $\tau_{1},\tau_{2},\ldots ,\tau_{\lambda }$, as well as 2(*m *- λ - 1) other 2-cycles $f_{\lambda +1},f_{\lambda +1}^{\prime },\ldots ,f_{m-1},f_{m-1}^{\prime }$ that cannot derive any good fusions to act on *π *since their T and C3 elements lie in the same contig strand in $\hat{\pi }$. If we can further extract two 2-cycles, say *f *and its mate 2-cycle *f*', from $\hat{\sigma }{\hat{\pi }}^{-1}$ that can derive a good fusion, say *τ*, to act on *π*, then the C3 elements in both *f *and *f*' must locate at a contig whose T elements are in some *f_k _*and $f_{k}^{\prime }$, respectively, where 1 ≤ *k *≤ λ. This implies that the good fusion *τ *cannot act on $\hat{\pi }$ together with $\tau_{1},\tau_{2},\ldots ,\tau_{\lambda }$ at the same time, since they will assemble a circular contig that is not allowed. Now, we suppose that $\tau_{1},\tau_{2},\ldots ,\tau_{m-1}$ are the fusions obtained by the step 4 of Algorithm 1. Clearly, for 1≤k≤λ, $||\psi \left(\hat{\sigma }{\hat{\omega }}_{k-1}^{-1}\right)||-||\psi \left(\hat{\sigma }{\hat{\omega }}_{k}^{-1}\right)||\;=2$ since *τ_k _*is a good fusion to $\omega_{k-1}$. Moreover, for $\lambda +1\leq k\leq m-1,\;||\psi \left(\hat{\sigma }{\hat{\omega }}_{k}^{-1}\right)||-||\psi \left(\hat{\sigma }{\hat{\omega }}_{k-1}^{-1}\right)||\;=0$, due to the following reason. According to Algorithm 1, we have $\tau_{k}=({\hat{\omega }}_{k-1}\hat{\text{Γ}}\left(z_{k}\right)$, ${\hat{\omega }}_{k-1}\hat{\text{Γ}}\left(x_{k}\right))({\hat{\omega }}_{k-1}\hat{\text{Γ}}\left(z_{k}\right)$, ${\hat{\omega }}_{k-1}\hat{\text{Γ}}\left(y_{k}\right))\left(y_{k},\;z_{k}\right)\left(x_{k},\;z_{k}\right)$, which actually equals to $({\hat{\omega }}_{k-1}\hat{\text{Γ}}\left(x_{k}\right)$, ${\hat{\omega }}_{k-1}\hat{\text{Γ}}\left(z_{k}\right)$, ${\hat{\omega }}_{k-1}\hat{\text{Γ}}\left(y_{k}\right))\left(y_{k},\;z_{k},\;x_{k}\right)$. Moreover, we have $\psi \left(\hat{\sigma }{\hat{\omega }}_{k}^{-1}\right)=\psi \left(\hat{\sigma }{\hat{\omega }}_{k-1}^{-1}\right)\tau_{k}^{-1}$, in which the composition of $\left(x_{k},\;y_{k}\right){\left(y_{k},\;z_{k},\;x_{k}\right)}^{-1}$ equals to $\left(x_{k},\;z_{k}\right)$ and the composition of $({\hat{\omega }}_{k-1}\hat{\text{Γ}}\left(y_{k}\right)$, ${\hat{\omega }}_{k-1}\hat{\text{Γ}}\left(x_{k}\right))({\hat{\omega }}_{k-1}\hat{\text{Γ}}\left(x_{k}\right)$, ${\hat{\omega }}_{k-1}\hat{\text{Γ}}\left(z_{k}\right)$, ${\hat{\omega }}_{k-1}\hat{\text{Γ}}\left(y_{k}\right){}^{)-1}$ equals to $({\hat{\omega }}_{k-1}\hat{\text{Γ}}\left(y_{k}\right),{\hat{\omega }}_{k-1}\hat{\text{Γ}}\left(z_{k}\right))$. Recall that $f_{k}=\left(x_{k},\;y_{k}\right)$ and $f_{k^{\prime }}=({\hat{\omega }}_{k-1}\hat{\text{Γ}}\left(y\right),{\hat{\omega }}_{k-1}\hat{\text{Γ}}\left(x\right))$, both of which are extracted from two mate cycles in $\psi \left(\hat{\sigma }{\hat{\omega }}_{k-1}^{-1}\right)$. According to the above discussion, both *y_k _*and $\hat{\pi }\hat{\text{Γ}}\left(x_{k}\right)$ will be fixed in $\psi \left(\hat{\sigma }{\hat{\omega }}_{k}^{-1}\right)$, thus increasing the number of cycles by two. However, the 2-cycle $\left(x_{k},\;z_{k}\right)$ will further join other two cycles respectively containing *x_k _*and *z_k _*together into one cycle and $(\hat{\pi }\hat{\text{Γ}}\left(y_{k}\right),\;\hat{\pi }\hat{\text{Γ}}\left(z_{k}\right))$ will join another two cycles respectively containing $\hat{\pi }\hat{\text{Γ}}\left(y_{k}\right)$ and $\hat{\pi }\hat{\text{Γ}}\left(z_{k}\right)$ together into one cycle, thus decreasing the number of cycles by two. As a result, $n_{c}(\psi \left(\hat{\sigma }{\hat{\omega }}_{k}^{-1}\right))=n_{c}(\psi \left(\hat{\sigma }{\hat{\omega }}_{k-1}^{-1}\right))$. Therefore, we have $||\psi \left(\hat{\sigma }{\hat{\omega }}_{0}^{-1}\right)||-||\psi \left(\hat{\sigma }{\hat{\omega }}_{m-1}^{-1}\right)||\;\leq 2\lambda$ for the (*m *-1) fusions obtained by the step 4 of Algorithm 1. In fact, to let $||\psi \left(\hat{\sigma }{\hat{\omega }}_{0}^{-1}\right)||-||\psi \left(\hat{\sigma }{\hat{\omega }}_{m-1}^{-1}\right)||\;>2\lambda$ happen, there must be a translocation *τ_i _*that acts on ${\hat{\omega }}_{i-1}$ as a fusion of $\omega_{i-1}$ satisfying either (1) $||\psi \left(\hat{\sigma }{\hat{\omega }}_{i-1}^{-1}\right)||-||\psi \left(\hat{\sigma }{\hat{\omega }}_{i}^{-1}\right)||\;=0$, the number of good fusions newly created by *τ_i _*and its following cap exchanges minus that of good fusions currently destroyed by *τ_i _*and the following cap exchanges is greater than or equal to one, and the total available good fusions can assemble more contigs than before, or (2) $||\psi \left(\hat{\sigma }{\hat{\omega }}_{i-1}^{-1}\right)||-||\psi \left(\hat{\sigma }{\hat{\omega }}_{i}^{-1}\right)||\;=-2$, the number of good fusions created by *τ_i _*and its following cap exchanges minus that of the currently destroyed good fusions is greater than or equal to two, and the total good fusions can assemble more contigs than before. However, we show below that no such a translocation *τ_i _*exits. Let $\tau_{i}=({\hat{\omega }}_{i-1}\hat{\text{Γ}}(5\text{cap}\left(y,\;{\hat{\omega }}_{i-1}\right))$, ${\hat{\omega }}_{i-1}\hat{\text{Γ}}(5\text{cap}\left(x,\;{\hat{\omega }}_{i-1}\right)))({\hat{\omega }}_{i-1}\hat{\text{Γ}}\left(y\right),\;{\hat{\omega }}_{i-1}\hat{\text{Γ}}\left(x\right))(5\text{cap}\left(x,\;{\hat{\omega }}_{i-1}\right)$, $5\text{cap}\left(y,\;{\hat{\omega }}_{i-1}\right))\left(x,\;y\right)$ be a fusion (but not a good one) to $\omega_{i-1}$, where char $\left(x,\;{\hat{\omega }}_{i-1}\right)=\text{T}$ and char $\left(y,\;{\hat{\omega }}_{i-1}\right)=\text{C}3$. According to Corollary 1, we have $\left(x,\;y\right)∤\hat{\sigma }{\hat{\omega }}_{i-1}^{-1}$, that is, *x *and *y *are in different cycles of $\hat{\sigma }{\hat{\omega }}_{i-1}^{-1}$. Moreover, char $\left(x,\;\tau_{i}{\hat{\omega }}_{i-1}\right)=\text{O}$ and char $\left(y,\;\tau_{i}{\hat{\omega }}_{i-1}\right)=\text{N}3$ after applying *τ_i _*to ${\hat{\omega }}_{i-1}$. Below, we consider two cases.

Case 1: Suppose that there is a 2-cycle $f_{j}=\left(x_{j},\;y_{j}\right)$ such that $x_{j}=x$, where 1 ≤j≤m-1, char $\left(x_{j},\;{\hat{\omega }}_{i-1}\right)=\text{T}$ and char $\left(y_{j},\;{\hat{\omega }}_{i-1}\right)=\text{C}3$. For simplifying our discussion, we assume that *f_j _*is disjoint from the other cycles in $\psi \left(\hat{\sigma }{\hat{\omega }}_{i-1}^{-1}\right)$ and *y *is in the cycle $\alpha =\left(a_{1},\;a_{2},\;\ldots ,\;a_{h}\equiv y\right)$ of $\psi \left(\hat{\sigma }{\hat{\omega }}_{i-1}^{-1}\right)$. Then in $\psi \left(\hat{\sigma }{\hat{\omega }}_{i-1}^{-1}\right)\tau_{i}^{-1}$, the cycles *f_j _*and *α *are joined into a cycle $\beta =\left(a_{1},\;a_{2},\;\ldots ,\;a_{h-1},\;y,\;y_{j},\;x\right)$, which can be expressed as $\gamma \left(y,\;y_{j}\right)$, where $\gamma =\left(a_{1},\;a_{2},\;\ldots ,\;a_{h-1},\;y,\;x\right)$, char $\left(y,\;\tau_{i}{\hat{\omega }}_{i-1}\right)=\text{N}3$ and char $\left(y_{j},\;\tau_{i}{\hat{\omega }}_{i-1}\right)=\text{C}3$. According to Lemma 3, there is a cycle $\beta^{\prime }=\left(\tau_{i}{\hat{\omega }}_{i-1}\hat{\text{Γ}}\right)\cdot \beta^{-1}$. that is the mate cycle of *β *in $\psi \left(\hat{\sigma }{\hat{\omega }}_{i-1}^{-1}\right)\tau_{i}^{-1}$. In other words, we can extract *c*_1 _= (*y*, *y_j_*) from *β *and $c_{1}^{\prime }=(\tau_{i}{\hat{\omega }}_{i-1}\hat{\text{Γ}}\left(y_{j}\right)$, $\tau_{i}{\hat{\omega }}_{i-1}\hat{\text{Γ}}\left(y\right))$ from $\beta^{\prime }$, and then apply $\tau_{i}^{\prime }=c_{2}^{\prime }c_{1}^{\prime }c_{2}c_{1}$ to $\tau_{i}{\hat{\omega }}_{i-1}$ as a cap exchange, where $c_{2}=(5\text{cap}\left(y,\;\tau_{i}{\hat{\omega }}_{i-1}\right),\;5\text{cap}\left(y_{j},\;\tau_{i}{\hat{\omega }}_{i-1}\right))$ and $c_{2}^{\prime }=(\tau_{i}{\hat{\omega }}_{i-1}\hat{\text{Γ}}(5\text{cap}\left(y_{j},\;\tau_{i}{\hat{\omega }}_{i-1}\right))$, $\tau_{i}{\hat{\omega }}_{i-1}\hat{\text{Γ}}(5\text{cap}\left(y,\;\tau_{i}{\hat{\omega }}_{i-1}\right)))$, since the character pair (C3, N3) of (*y_j_*, *y*) belongs to CEpair. After that, *y_j_*, as well as $\tau_{i}{\hat{\omega }}_{i-1}\hat{\text{Γ}}\left(y\right)$, will be fixed in the resulting $\psi \left(\hat{\sigma }{\hat{\omega }}_{i}^{-1}\right)$ and char $\left(y,\;{\hat{\omega }}_{i}\right)$ will become C3. As a result, $n_{c}(\psi \left(\hat{\sigma }{\hat{\omega }}_{i}^{-1}\right))=n_{c}(\psi \left(\hat{\sigma }{\hat{\omega }}_{i-1}^{-1}\right))$ and hence $||\psi \left(\hat{\sigma }{\hat{\omega }}_{i-1}^{-1}\right)||-||\psi \left(\hat{\sigma }{\hat{\omega }}_{i}^{-1}\right)||\;=0$. According to the above discussion, if *j *≤ λ, that is, *f_j _*cannot be used to derive a good fusion to $\omega_{i-1}$, then acting $\tau_{i}^{\prime }\tau_{i}$ on ${\hat{\omega }}_{i-1}$ still serves as a fusion of $\omega_{i-1}$ and after that, it can be verified that no existing good fusion is destroyed and no new good fusion is created. If *j *≤ λ, that is, *f_j _*can be used to derive a good fusion to $\omega_{i-1}$, then this good fusion will be destroyed when we perform $\tau_{i}^{\prime }\tau_{i}$ on ${\hat{\omega }}_{i-1}$. Suppose that char$\left(a_{h-1},\;{\hat{\omega }}_{i-1}\right)=\text{T}$. Then after further performing the cap exchange $\tau_{i}^{\prime }$ on $\tau_{i}{\hat{\omega }}_{i-1}$, we still can extract a 2-cycle (*a*_*h*-1_, *y*) from γ with character pair of (T, C3) in the resulting ${\hat{\omega }}_{i}$. Clearly, if (*a*_*h*-1_, *y*) = *f_k _*with *k *< λ, that is, *f_k _*cannot derive a good fusion to *ω*_*i*-1_ (*a*_*h*-1 _and *y *are in the same cycle of ${\hat{\omega }}_{i-1}$), then after performing the cap exchange $\tau_{i}^{\prime }$ on $\tau_{i}{\hat{\omega }}_{i-1}$, it can be used to derive a good fusion to ${\hat{\omega }}_{i}$, since *a_h-1 _*and *y *will be separated by $\tau_{i}^{\prime }$ into two different cycles in the resulting ${\hat{\omega }}_{i}$. If *k *≤ λ, that is, *f_k _*can derive a good fusion to *ω*_*i*-1_, then after performing the cap exchange $\tau_{i}^{\prime }$ on $\tau_{i}{\hat{\omega }}_{i-1}$, *f_k _*can or cannot derive a good fusion to ${\hat{\omega }}_{i}$. Based on the above discussion, the number of good fusions newly created by *τ_i _*and $\tau_{i}^{\prime }$ minus that of good fusions currently destroyed by *τ_i _*and $\tau_{i}^{\prime }$ must be less than or equal to zero.

Case 2: Suppose that there is no $f_{j}=\left(x_{j},\;y_{j}\right)$ such that *x_j _*= *x*, where 1≤j≤m-1, char $\left(x_{j},{\hat{\omega }}_{i-1}\right)=\text{T}$ and char $\left(y_{j},{\hat{\omega }}_{i-1}\right)=\text{C}3$. Let *α*_1 _denote the cycle containing *x *and *α*_2 _denote the cycle containing *y *in $\hat{\sigma }{\hat{\omega }}_{i-1}^{-1}$. Also let $\alpha_{1}^{\prime }$ and $\alpha_{2}^{\prime }$ be the mate cycles of *α*_1 _and *α*_2_, respectively, in $\hat{\sigma }{\hat{\omega }}_{i-1}^{-1}$. Note that after applying *τ_i _*to ${\hat{\omega }}_{i-1}$, the cycles *α*_1 _and *α*_2 _will be merged into a single cycle, say *α*, in $\hat{\sigma }{\hat{\omega }}_{i-1}^{-1}\tau_{i}^{-1}$ and $\alpha_{1}^{\prime }$ and $\alpha_{2}^{\prime }$ will be merged into a single cycle, say $\alpha^{\prime }$. Moreover, the characters of *x *and *y *in $\tau_{i}{\hat{\omega }}_{i-1}$ will become O and N3, respectively. As discussed in the proof of Lemma 6, if both *α*_1 _and *α*_2_, as well as both $\alpha_{1}^{\prime }$ and $\alpha_{2}^{\prime }$, do not contain T and C3 elements simultaneously, then $||\psi \left(\hat{\sigma }{\hat{\omega }}_{i-1}^{-1}\right)||-||\psi \left(\hat{\sigma }{\hat{\omega }}_{i}^{-1}\right)||\;=-2$. In this case, it can be verified that no existing good fusion is destroyed by *τ_i _*and no new good fusion is created by *τ_i_*. In other words, the number of the increased good fusions minus that of the destroyed good fusions is zero. If at least one of *α*_1 _and *α*_2_, as well as at least one of $\alpha_{1}^{\prime }$ and $\alpha_{2}^{\prime }$, has both T and C3 elements at the same time, then $||\psi \left(\hat{\sigma }{\hat{\omega }}_{i-1}^{-1}\right)||-||\psi \left(\hat{\sigma }{\hat{\omega }}_{i}^{-1}\right)||\;=0$ according to the discussion in the proof of Lemma 6. Now suppose that *α*_1 _has no C3 element. Then T and C3 elements in *α*_2 _can form a 2-cycle that equals to some $f_{k}=\left(x_{k},\;y_{k}\right)$, where 1≤k≤m-1 and $y_{k}=y$. After applying *τ_i _*to ${\hat{\omega }}_{i-1}$, the T element *x *from *α*_1 _becomes an O element in *α *and the C3 element *y *from *α*_2 _becomes an N3 element in *α*. We can continue to extract $\left(x_{k},\;y\right)$, which is now a 2-cycle of (T, N3), from *α *and act $\tau_{i}^{\prime }=c_{2}^{\prime }c_{1}^{\prime }c_{2}c_{1}$ on $\tau_{i}{\hat{\omega }}_{i-1}$ as a cap exchange, where $c_{1}=\left(x_{k},\;y\right)$, $c_{2}=(5\text{cap}\left(x_{k},\;\tau_{i}{\hat{\omega }}_{i-1}\right)$, $5\text{cap}\left(y,\;\tau_{i}{\hat{\omega }}_{i-1}\right))$, $c_{1}^{\prime }=(\tau_{i}{\hat{\omega }}_{i-1}\hat{\text{Γ}}\left(y\right),\;\tau_{i}{\hat{\omega }}_{i-1}\hat{\text{Γ}}\left(x_{k}\right))$ and $c_{2}^{\prime }=(\tau_{i}{\hat{\omega }}_{i-1}\hat{\text{Γ}}(5\text{cap}\left(y,\;\tau_{i}{\hat{\omega }}_{i-1}\right))$, $\;\tau_{i}{\hat{\omega }}_{i-1}\hat{\text{Γ}}(5\text{cap}\left(x_{k},\;\tau_{i}{\hat{\omega }}_{i-1}\right)))$. Clearly, no new good fusion is created in this case and one existing good fusion derived by *f_k _*will be destroyed if k≤λ. Therefore, the number of the increased good fusions minus that of the destroyed good fusions is less than or equal to zero. Suppose that *α*_1 _contains both T and C3 elements, where we denote the C3 element by *z *for convenience. Then *x *and *z *can form a 2-cycle of (T, C3) pair, which can derive a good fusion $\tau =c_{2}^{\prime }c_{1}^{\prime }c_{2}c_{1}$ to ${\hat{\omega }}_{i-1}$ if $\left(x,\;z\right)∤{\hat{\omega }}_{i-1}$, where $c_{1}=\left(x,\;z\right)$, $c_{2}=(5\text{cap}\left(x,{\hat{\omega }}_{i-1}\right)$, $5\text{cap}\left(z,{\hat{\omega }}_{i-1}\right))$, $c_{1}^{\prime }=({\hat{\omega }}_{i-1}\hat{\text{Γ}}\left(z\right),{\hat{\omega }}_{i-1}\hat{\text{Γ}}\left(x\right))$ and $c_{2}^{\prime }=({\hat{\omega }}_{i-1}\hat{\text{Γ}}(5\text{cap}\left(z,{\hat{\omega }}_{i-1}\right))$, ${\hat{\omega }}_{i-1}\hat{\text{Γ}}(5\text{cap}\left(x,{\hat{\omega }}_{i-1}\right)))$. If $\left(x,\;z\right)∤{\hat{\omega }}_{i-1}$, then, as mentioned previously, *τ *cannot work together with *λ *other good fusions derived by $f_{1},f_{2},\ldots ,f_{\lambda }$ at the same time, since they will assemble a circular contig that is not allowed. For the case in which *α*_2 _contains no T element, it is not hard to see that no new good fusion will be created and no existing good fusion will be destroyed when performing $\tau_{i}$ and its following cap exchange on $\omega_{i-1}$, resulting in that the number of the created good fusions minus that of the destroyed good fusions is zero. We now assume that $\alpha_{2}$ contains a T element, say *w*, and a C3 element *y*. Then *w *and *y *are adjacent in $\alpha_{2}$ and (*w*, *y*) equals to some *f_k_*, where 1≤k≤m-1. After applying $\tau_{i}$ to ${\hat{\omega }}_{i-1},\alpha$ has a C3 element *z*, a T element *w *and an N3 element *y*. Then a 2-cycle (*w*, *y*) can be extracted from *α *such that $\tau_{i}^{\prime }=c_{2}^{\prime }c_{1}^{\prime }c_{2}c_{1}$ can further perform on $\tau_{i}{\hat{\omega }}_{i-1}$ as a cap exchange, where $c_{1}=\left(w,\;y\right)$, $c_{2}=(5\text{cap}\left(w,\;\tau_{i}{\hat{\omega }}_{i-1}\right)$, $5\text{cap}\left(y,\;\tau_{i}{\hat{\omega }}_{i-1}\right))$, $c_{1}^{\prime }=(\tau_{i}{\hat{\omega }}_{i-1}\hat{\text{Γ}}\left(y\right),\;\tau_{i}{\hat{\omega }}_{i-1}\hat{\text{Γ}}\left(w\right))$ and $c_{2}^{\prime }=(\tau_{i}{\hat{\omega }}_{i-1}\hat{\text{Γ}}(5\text{cap}\left(y,\;\tau_{i}{\hat{\omega }}_{i-1}\right))$, $\tau_{i}{\hat{\omega }}_{i-1}\hat{\text{Γ}}(5\text{cap}\left(w,\;\tau_{i}{\hat{\omega }}_{i-1}\right)))$. Hence, if k≤λ, then the good fusion derived by *f_k _*will be destroyed by *τ_i _*and $\tau_{i}^{\prime }$. However, the remaining *α *still contains a C3 element *z *and a T element *w*, which can derive a good fusion, say $\tau^{\prime }$. Hence, the number of the increased good fusions minus that of the destroyed good fusions is zero. On the other hand, if k > λ, then no exiting good fusion is destroyed by *τ_i _*and $\tau_{i}^{\prime }$. In this case, the number of the increased good fusions minus that of the destroyed good fusions is equal to one. However, it can be verified that $\tau^{\prime }$ cannot work with *λ *other good fusions derived by $f_{1},f_{2},\ldots ,f_{\lambda }$, because they will produce a circular contig that is not allowed. In other words, no more contigs can be assembled after performing $\tau_{i}$ and $\tau_{i}^{\prime }$ on ${\hat{\omega }}_{i-1}$.

According to the above discussion, we can conclude that $||\psi \left(\hat{\sigma }{\hat{\omega }}_{0}^{-1}\right)||-||\psi \left(\hat{\sigma }{\hat{\omega }}_{m-1}^{-1}\right)||\;\leq 2\lambda$.    □

Based on Lemma 7, as well as the discussion in its proof, the *m *- 1 fusions derived by Algorithm 1 correspond to an optimal ordering of *π *with an induced permutation *assembly *(*π*) such that the weighted rearrangement distance Δ(π,σ) between *assembly *(*π*) and *σ *is minimized. The obtained rearrangement distance Δ(π,σ) is calculated based on the algorithm in our previous study [17], and is equal to $\frac{\left||\hat{\sigma }{\hat{\pi }}^{-1}|\right|}{2}$, where $\hat{\pi }$ is the genome obtained by performing the cap exchanges and *m *- 1 fusions on the initial capping of *π*, as done in the steps 3 and 4 in Algorithm 1, respectively. The total time complexity of Algorithm 1 is O(δn), where *δ *is the number of reversals and block-interchanges used to transform *assembly *(*π*) into *σ*. The reason is as follows. Since m≤n, the cost of the step 1 for capping the input genomes is O(n). The computation of $\hat{\sigma }{\hat{\pi }}^{-1}$ in the step 2 still can be done in O(n) time. Recall that after running the step 3, each cycle in $\hat{\sigma }{\hat{\pi }}^{-1}$ has at most a  T element and at most a C3 element. Totally, there are 2mT elements and 2*m *C3 elements in the cycles of $\hat{\sigma }{\hat{\pi }}^{-1}$. Moreover, deriving two 2-cycles to serve as a cap exchange from two long mate cycles in $\hat{\sigma }{\hat{\pi }}^{-1}$ will divide these two long cycles into four smaller cycles. Hence, there are O(n) cap exchanges to be performed in the step 3, which totally cost O(n) time since each cap exchange needs only constant time. The step 4 assembles *m *contigs by utilizing 2(*m *- 1) 2-cycle $f_{1},f_{1}^{\prime },\ldots ,f_{m-1},f_{m-1}^{\prime }$, which can be derived in advance from $\hat{\sigma }{\hat{\pi }}^{-1}$ in O(n) time. Since each fusion requires only constant time, the cost of the step 4 is O(m+n), which is equal to O(n). As to the steps 5 and 6, they can be done in O(δn) time in total, since there are totally *δ *iterations to find the reversals and block-interchanges and the time complexity of each iteration is dominated by the cost of finding a reversal or block-interchange that is O(n) time. Notice that although Algorithm 1 we described above is dedicated to linear, uni-chromosomal genomes, it can still be applied to circular, uni-chromosomal genomes, or to multi-chromosomal genomes with linear or circular chromosomes in a way of chromosome by chromosome.

**Theorem 1 ***Given a partially assembled genome π and a completely assembled genome σ, the one-sided block ordering problem can be solved in O*(*δn*) *time and the weighted rearrangement distance between the permutation assembly*(*π*) *induced by the optimal ordering of π and σ is *$\frac{\left||\hat{\sigma }{\hat{\pi }}^{-1}|\right|}{2}$*that can be computed in O*(*n*) *time, where *$\hat{\pi }$*is the capping genome of π with the cap exchanges and m - *1 *fusions being done, *$\hat{\sigma }$*is the capping genome of σ, n is the number of genes or markers, and δ is the number of reversals and block-interchanges used to transform assembly*(*π*) *into σ*.

As mentioned in the introduction, any algorithm to solve the one-sided block ordering problem can be used to assemble (i.e., order and orient) the contigs in a draft genome based on a reference genome, if we denote this draft genome as *π *and use the reference genome as *σ*. For this application, our Algorithm 1 can finish its job just in O(n) time, because it does not need to do the steps 5 and 6 in this situation.

## Experimental results

**Figure 1:**
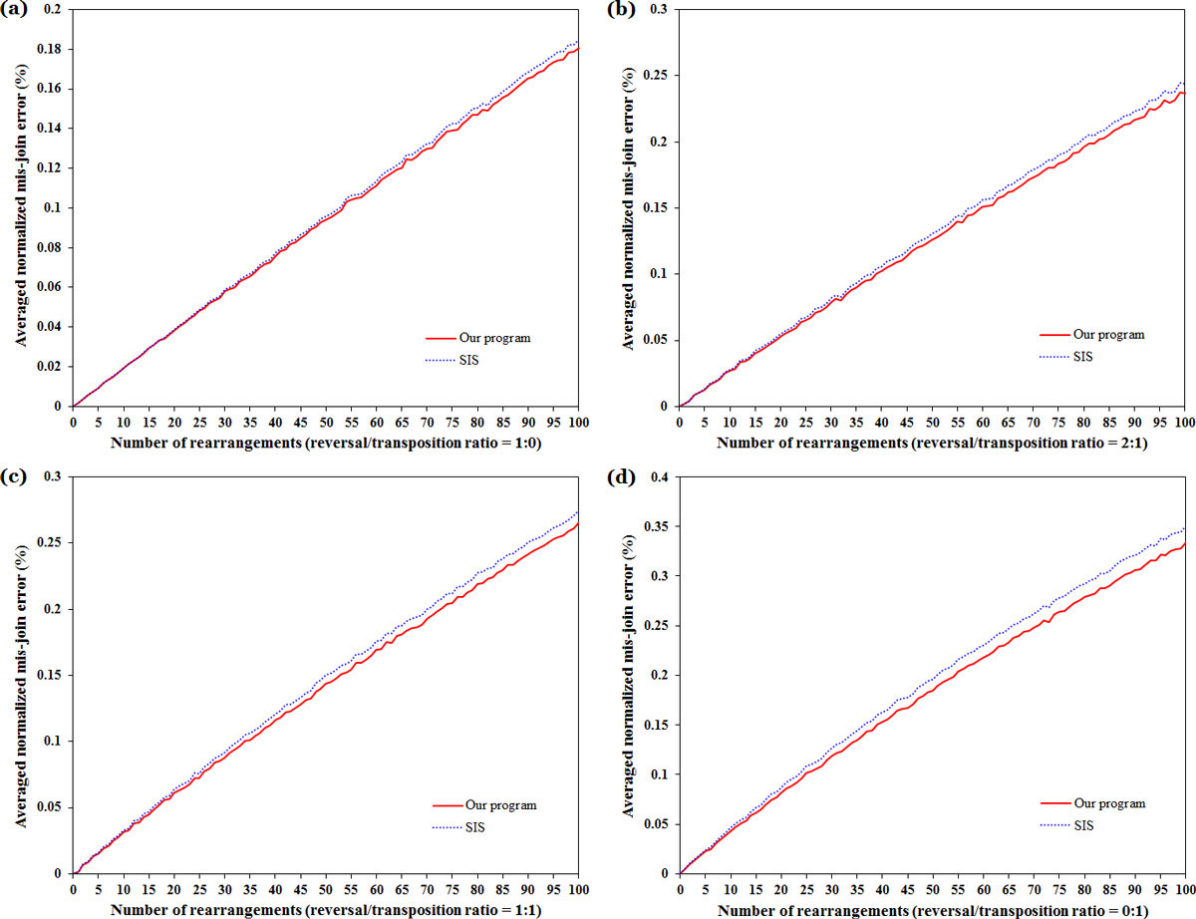
**Comparison of accuracy performance between our program and SIS on simulated datasets with different ratios of the involved reversals and transpositions**.

We have implemented Algorithm 1 as mentioned in the previous section into a program and also compared its accuracy performance to SIS on assembling the contigs of partially assembled genomes using some simulated datasets of linear, uni-chromosomal genomes. For this purpose, we compared the permutation induced by an assembly algorithm for a partially assembled genome with its actual permutation by counting the number of breakpoints between them, where each breakpoint corresponds to an error of incorrectly joining two contigs (i.e., a mis-join error) caused by the assembly algorithm. This breakpoint number is then normalized by the number of contigs minus *ρ *to represent a fraction of incorrect contig joins, where *ρ *= 1 if the chromosome is linear; otherwise, *ρ *= 0. Each of partially assembled genomes with single linear chromosome in our simulated datasets was prepared and tested as follows. First, we generated the reference genome *σ *= (1, 2, ..., *n*) with a linear chromosome of *n *genes, where *n *varies from 50 to 1000 with in the step of 50, and performed *δ *random rearrangement events (reversals and/or transpositions) on *s *to obtain a permutation of a linear, uni-chromosomal genome *π*', where *δ *varies from zero to 100 in the step of 1. Among the *δ *rearrangement events in our simulations, we used four different occurrence ratios to randomly generate reversals and transpositions: (1) 1:0, (2) 2:1, (3) 1:1 and (4) 0:1. Next, the genome $\pi^{\prime }$ is randomly fragmented into *m *contigs of various sizes to simulate the partially assembled genome *π*, where *m *varies from 50 to 500 with step 50. Finally, for each choice of *n, m, δ *and reversal/transposition ratio, we repeated the experiments 10 times and compared our program with SIS using their averaged normalized mis-join errors. As shown in Figure 1, the averaged normalized contig mis-join errors of our program are lower than those of SIS for all simulated datasets when the number of the involved reversals and transpositions is increased. In particular, if there are more transpositions involved in the rearrangement events, then the gap of accuracy performance between our program and SIS is increasing. The main reason may be due to the fact that our program can deal with both reversals and block-interchanges (including transpositions as a special case), while SIS considers only reversals without taking into account transpositions.

## Conclusions

In this study, we introduced and studied the one-sided block/contig problem with optimizing the weighted reversal and block-interchange distance, which particularly has a useful application in genome resequencing. We finally designed an efficient algorithm to solve this problem in O(δn) time, where *n *is the number of genes or markers and *d *is the number of used reversals and block-interchanges. In addition, we showed that the assembly of the partially assembled genome can be done in O(n) time and its weighted rearrangement distance from the completely assembled genome can be calculated in advance in O(n) time. Finally, our simulation results showed that the accuracy performance of our program is better than that of the currently existing tool SIS when the number of the involved reversals and transpositions is increased. Moreover, the gap of this accuracy performance between our program and SIS is increasing, if there are more transpositions involved in the rearrangement events.

## Competing interests

The authors declare that they have no competing interests.

## Authors' contributions

Corresponding author CLL conceived of the study, designed and analyzed the algorithm, and drafted the manuscript. The other authors CLL and KTC participated in the development of the program, as well as in the simulated experiments and their result discussion. The authors wish it to be known that the first two authors CLL and KTC contributed equally to this work and should be considered co-first authors. All authors read and approved the final manuscript.

## Declarations

The publication costs for this article were funded by National Science Council of Republic of China under grant NSC100-2221-E-007-129-MY3.

This article has been published as part of *BMC Bioinformatics *Volume 14 Supplement 5, 2013: Proceedings of the Third Annual RECOMB Satellite Workshop on Massively Parallel Sequencing (RECOMB-seq 2013). The full contents of the supplement are available online at http://www.biomedcentral.com/bmcbioinformatics/supplements/14/S5.

## References

[B1] Shendure J Ji HL Next-generation DNA sequencing Nature Biotechnology 2008 26 1135 1145 10.1038/nbt1486 18846087

[B2] Mardis ER The impact of next-generation sequencing technology on genetics Trends in Genetics 2008 24 133 141 10.1016/j.tig.2007.12.007 18262675 PMC2680276

[B3] Metzker ML Sequencing technologies - the next generation Nature Reviews Genetics 2010 11 31 46 10.1038/nrg2626 19997069

[B4] Fertin G Labarre A Rusu I Tannier E Vialette S Combinatorics of Genome Rearrangements 2009 Cambridge, Massachusetts: The MIT Press

[B5] Gaul E Blanchette M Ordering partially assembled genomes using gene arrangements Lecture Notes in Computer Science 2006 4205 113 128 10.1007/11864127_10

[B6] Bourque G Pevzner PA Genome-scale evolution: reconstructing gene orders in the ancestral species Genome Research 2002 12 26 36 11779828 PMC155248

[B7] Hannenhalli S Pevzner PA Transforming cabbage into turnip: polynomial algorithm for sorting signed permutations by reversals Journal of the ACM 1999 46 1 27 10.1145/300515.300516

[B8] Bentley DR Whole-genome re-sequencing Current Opinion in Genetics and Development 2006 16 545 552 10.1016/j.gde.2006.10.009 17055251

[B9] Koboldt DC Ding L Mardis ER Wilson RK Challenges of sequencing human genomes Briefings in Bioinformatics 2010 11 484 498 10.1093/bib/bbq016 20519329 PMC2980933

[B10] van Hijum SAFT Zomer AL Kuipers OP Kok J Projector 2: contig mapping for efficient gap-closure of prokaryotic genome sequence assemblies Nucleic Acids Research 2005 33 W560 W566 10.1093/nar/gki356 15980536 PMC1160117

[B11] Richter DC Schuster SC Huson DH OSLay: optimal syntenic layout of unfinished assemblies Bioinformatics 2007 23 1573 1579 10.1093/bioinformatics/btm153 17463020

[B12] Assefa S Keane TM Otto TD Newbold C Berriman M ABACAS: algorithm-based automatic contiguation of assembled sequences Bioinformatics 2009 25 1968 1969 10.1093/bioinformatics/btp347 19497936 PMC2712343

[B13] Rissman AI Mau B Biehl BS Darling AE Glasner JD Perna NT Reordering contigs of draft genomes using the Mauve Aligner Bioinformatics 2009 25 2071 2073 10.1093/bioinformatics/btp356 19515959 PMC2723005

[B14] Muñoz A Zheng CF Zhu QA Albert VA Rounsley S Sankoff D Scaffold filling, contig fusion and comparative gene order inference BMC Bioinformatics 2010 11 304 10.1186/1471-2105-11-304 20525342 PMC2902449

[B15] Husemann P Stoye J r2cat: synteny plots and comparative assembly Bioinformatics 2010 26 570 571 10.1093/bioinformatics/btp690 20015948 PMC2820676

[B16] Dias Z Dias U Setubal JC SIS: a program to generate draft genome sequence scaffolds for prokaryotes BMC Bioinformatics 2012 13 96 10.1186/1471-2105-13-96 22583530 PMC3674793

[B17] Huang YL Lu CL Sorting by reversals, generalized transpositions, and translocations using permutation groups Journal of Computational Biology 2010 17 685 705 10.1089/cmb.2009.0025 20500022

[B18] Blanchette M Kunisawa T Sankoff D Parametric genome rearrangement Gene 1996 172 GC11 GC17 10.1016/0378-1119(95)00878-0 8654963

